# DCR-like restrictive resuscitation is associated with enhanced oxygen metabolism, coagulation recovery, and reduced inflammation in severe chest trauma and shock

**DOI:** 10.3389/fphys.2026.1827424

**Published:** 2026-07-15

**Authors:** Zhi Hu, Tingzhong Wang, Yuejiao Wei, Xiaolin Hou, Qiang Song

**Affiliations:** 1 Department of Cardiovascular Medicine, The First Affiliated Hospital of Xi’an Jiaotong University, Xi’an, China; 2 Department of Cardiovascular Medicine, Sichuan Academy of Medical Sciences and Sichuan Provincial People’s Hospital (Affiliated Hospital of University of Electronic Science and Technology of China), Sichuan, Chengdu, China

**Keywords:** chest trauma, coagulation, fluid resuscitation, hemorrhagic shock, inflammation, oxygen metabolism, propensity score matching, restrictive strategy

## Abstract

**Background:**

The optimal fluid resuscitation strategy for severe chest trauma with hemorrhagic shock remains debated, with limited evidence incorporating comprehensive physiological endpoints. This study compared a restrictive (damage-control resuscitation-like) strategy versus conventional crystalloid-predominant resuscitation in this high-risk population.

**Methods:**

This single-center retrospective cohort study used propensity score matching (1:1) to balance age, sex, Injury Severity Score, and shock severity, yielding 200 matched patients (100 per group). Outcomes included 28-day survival, major complications (ARDS, DIC, MODS), ventilator- and ICU-free days, and serial (0–72 h) assessments of oxygen metabolism (SvO_2_, DO_2_, VO_2_, lactate), coagulation (APTT, PT, HCT), and inflammatory markers (IL-6, IL-10, TNF-α). Exploratory composite metrics (Oxygen Debt Index, Coagulation Recovery Rate, Inflammation Burden Score) were also evaluated.

**Results:**

The restrictive group received significantly less total fluid (1985 ± 325 vs. 2846 ± 492 mL, *P* < 0.001) with higher fluid efficiency. It was associated with faster recovery in oxygen metabolism (lower ODI, *P* < 0.001, and higher lactate clearance), more rapid coagulation normalization (higher CRR and better APTT/PT), and steeper declines in inflammatory markers (IL-6, TNF-α) and IBS. Clinically, restrictive strategy was associated with higher 28-day survival (95% vs. 82%, *P* = 0.003), lower ARDS (16% vs. 34%), DIC (7% vs. 20%), and MODS (15% vs. 36%), and more ventilator- and ICU-free days. In multivariable analysis, restrictive resuscitation remained independently associated with lower 28-day mortality (HR = 0.23, 95% CI 0.09–0.58).

**Conclusion:**

In this observational, single-center study, a DCR-like restrictive strategy was associated with superior physiological trajectories and improved short-term clinical outcomes compared with conventional resuscitation. These hypothesis-generating findings do not establish causality and underscore the need for prospective randomized controlled trials before any practice change.

## 1 Introduction

Severe chest trauma is a life-threatening emergency frequently complicated by hemorrhagic shock, accounting for approximately 10–15% of all trauma cases and constituting a major cause of early mortality (Elkbuli et al., 2021; Bu et al., 2022; Jang et al., 2022; Mitchnik et al., 2022; Taniguchi et al., 2025). Hemorrhagic shock secondary to chest trauma leads to reduced circulating volume, tissue hypoperfusion, and oxygen debt, which may rapidly culminate in multiple organ dysfunction if resuscitation is delayed or inappropriate (Weng et al., 2019; Zhu et al., 2022; Yao et al., 2024). Beyond simple hypovolemia, traumatic hemorrhagic shock triggers microcirculatory dysfunction, oxygen metabolism disturbances, systemic inflammation, and coagulopathy—the so-called “lethal triad” of hypoperfusion, acidosis, and coagulopathy (Shao et al., 2019b; Weng et al., 2019; Jang et al., 2022).

Traditional fluid resuscitation has focused on rapid volume replacement using crystalloids or colloids (Wang et al., 2020; Zhang et al., 2022; Zhang et al., 2023). However, growing evidence highlights potential harms of aggressive crystalloid administration, including hemodilution, dilutional coagulopathy, tissue edema, and exacerbated inflammation, which may increase risks of acute lung injury, abdominal compartment syndrome, and multiple organ failure (Holcomb et al., 2015; Jiang et al., 2020; Yang et al., 2021; Anker et al., 2024). In response, the paradigm has shifted toward damage control resuscitation (DCR), which emphasizes permissive hypotension, early and balanced blood product use, and limited crystalloid exposure (Bickell et al., 1994). The landmark trial by Bickell et al. (1994) demonstrated that delayed, restricted fluid improved survival in penetrating torso injury, and subsequent studies have supported restrictive approaches in mixed trauma populations (Chi et al., 2022; Qian and Zhang, 2024). Current guidelines now recommend a restrictive volume strategy until definitive bleeding control is achieved (Guidelines for resuscitation of hypovolemic shock (2007), 2008).

Studies specifically in chest trauma have reported reduced ARDS and MODS with restrictive resuscitation (Weng et al., 2019; Jang et al., 2022). Recent evidence has further extended these findings: Li et al. (2026) (80 traumatic shock patients) showed improved blood gas, coagulation, and inflammatory markers with reduced complications; Zheng et al. (2023) (139 hemorrhagic shock patients) reported lower bleeding volume and enhanced coagulation recovery; and Shao et al. (2019a) found that targeting MAP 65–70 mmHg optimally suppressed pro-inflammatory cytokines. The 2025 ESICM guideline (Mekontso Dessap et al., 2025) now recommends a restrictive fluid strategy for blunt trauma hemorrhagic shock (moderate certainty). Despite this accumulating evidence, the application of restrictive resuscitation specifically in severe chest trauma remains an area of active investigation. Chest trauma presents unique challenges due to concomitant respiratory compromise and the heightened risk of ARDS. Comprehensive evaluations incorporating serial measurements of oxygen metabolism, coagulation trajectories, and inflammatory responses are still lacking for this population, and the optimal timing and targets require further elucidation.

It is critical to recognize that the restrictive strategy evaluated in this study encompasses multiple components of DCR—including limited crystalloid exposure, permissive hypotension, and earlier preferential use of blood products—rather than fluid volume reduction alone. Thus, the comparison is better characterized as evaluating a DCR-like restrictive strategy versus a conventional, crystalloid-predominant approach. This distinction is essential for interpreting the findings, as any observed differences may reflect the combined effects of multiple DCR elements rather than the independent contribution of DCR-like restrictive resuscitation strategy.

Therefore, this propensity score-matched retrospective cohort study aimed to compare a DCR-like restrictive resuscitation strategy versus conventional crystalloid-predominant resuscitation in severe chest trauma with hemorrhagic shock, using multi-time-point dynamic monitoring of oxygen metabolism (SvO_2_, DO_2_, VO_2_, lactate), coagulation (APTT, PT, HCT), and inflammatory markers (IL-6, IL-10, TNF-α). Exploratory composite metrics (Oxygen Debt Index, Coagulation Recovery Rate, Inflammation Burden Score) were also examined to summarize pathophysiological trajectories, with all interpretations framed in terms of strategy-level associations rather than isolated fluid effects.

## 2 Methods

### 2.1 Study design and ethical statement

This study was designed as a single-center retrospective comparative-effectiveness cohort study with propensity score matching. Consecutive adult patients with severe chest trauma complicated by hemorrhagic shock who were admitted to the Trauma Treatment Center of our hospital between June 2023 and December 2025 were screened. The protocol was reviewed and approved by the *First Affiliated Hospital of Xi ‘an Jiaotong University* Ethics Committee (Ethics No.: ZZJJBY25-07LC), and the requirement for individual informed consent was waived because of the retrospective design and anonymized data extraction.

### 2.2 Study population

Inclusion criteria were: (1) age ≥18 years; (2) admission diagnosis of severe chest trauma, defined as either (a) Chest Abbreviated Injury Scale (AIS) score ≥3, or (b) thoracic AIS ≥3, in combination with at least one of the following: multiple rib fractures (≥3 ribs) or flail chest, extensive pulmonary contusion (involving ≥2 lobes or >20% of lung volume on CT), massive hemothorax (>1,500 mL on initial drainage or persistent bleeding >200 mL/h), chest wall collapse, or major thoracic vascular injury requiring intervention; (3) hemorrhagic shock on presentation or during the immediate admission period; and (4) receipt of protocolized early resuscitation and monitoring in the trauma center. By focusing on this population, the study targeted a thoracic-dominant trauma phenotype in which fluid strategy may plausibly influence both circulatory rescue and pulmonary/inflammatory sequelae.

Exclusion criteria were: (1) severe preexisting coagulopathy or non-reversible long-term anticoagulation; (2) devastating craniocerebral injury (GCS ≤8 with primary risk of death from brain injury); (3) pregnancy, end-stage malignancy, or expected survival <24 h from non-thoracic causes; (4) incomplete key resuscitation records; and (5) transfer after major resuscitation had already been completed elsewhere. These criteria were chosen to enrich a clinically coherent cohort and reduce major competing sources of outcome heterogeneity.

Of 347 patients screened, 286 met eligibility criteria and were included in the original cohort according to the actual early resuscitation strategy documented in the medical record. PSM was performed to reduce treatment-selection bias using age, sex, ISS, and shock severity as covariates (caliper 0.2), yielding 100 matched pairs (200 patients) for the primary analysis.

### 2.3 Resuscitation strategies and treatment procedures

All patients were managed according to the institutional trauma emergency pathway upon admission. Airway stabilization, oxygenation/ventilatory support, rapid vascular access (at least two large-bore peripheral or central lines), invasive arterial blood pressure monitoring, and laboratory assessments (blood gas, lactate, coagulation panel, complete blood count) were performed in parallel. Urgent bleeding source identification was pursued via focused assessment with sonography in trauma (FAST), computed tomography angiography when hemodynamically feasible, or immediate operative exploration for exsanguinating patients.

The choice of resuscitation strategy was not randomized but was based on the attending trauma surgeon’s preference and the patient’s clinical presentation during the first 2 hours of resuscitation. Patients were retrospectively assigned to one of two groups according to the early fluid resuscitation protocol documented in the medical record, as defined below.

#### 2.3.1 Conventional resuscitation group (Conventional)

- Hemodynamic target: Mean arterial pressure (MAP) ≥ 65 mmHg or systolic blood pressure (SBP) ≥ 90 mmHg, aimed to be achieved within the first 30–60 minutes.
- Fluid protocol: Initial bolus of 500–1000 mL of isotonic crystalloid (lactated Ringer’s or normal saline) infused over 15–30 minutes, repeated if MAP remained <65 mmHg, with no predefined upper limit on total crystalloid volume before hemostatic control. Colloids (hydroxyethyl starch or albumin) were permitted at clinician discretion.
- Blood products: Red blood cell transfusion was triggered by hemoglobin <7 g/dL or ongoing hemorrhage with shock. Fresh frozen plasma and platelets were administered based on institutional massive transfusion protocol (usually 1:1:1 ratio after 4–6 units of red blood cells) but were not prioritized over crystalloid in the early phase.
- Termination of early resuscitation: Continued until MAP ≥65 mmHg sustained for 1 hour or until surgical bleeding control was achieved.

#### 2.3.2 Restrictive resuscitation group (Restrictive)

- Hemodynamic target: Permissive hypotension targeting MAP 50–65 mmHg (or SBP 70–90 mmHg) until definitive hemostasis was achieved. If traumatic brain injury was suspected (GCS <9 or abnormal head CT), MAP was maintained ≥80 mmHg per guidelines, and such patients were excluded from the restrictive group unless explicitly documented as having received permissive hypotension.
- Fluid protocol: Crystalloid limited to a maximum of 500–1000 mL total before hemostatic control. Bolus doses of 250 mL were given only if MAP fell below 50 mmHg or if signs of critical hypoperfusion (e.g., deteriorating mental status, thready pulse, lactate >4 mmol/L with rising trend) were present. No prophylactic fluid loading was permitted. Colloids were discouraged.
- Blood products: Early and preferential use of blood products (red blood cells, fresh frozen plasma, platelets) was encouraged to maintain hematocrit >21% and normalize coagulation parameters, aiming to avoid large crystalloid volumes. Transfusion thresholds were hemoglobin <8 g/dL or ongoing hemorrhagic shock.
- Termination of early resuscitation: Once bleeding was surgically controlled or the patient’s MAP spontaneously rose above 65 mmHg without ongoing vasopressor requirement.

Assignment to groups: A patient was classified into the conventional group if (a) the documented resuscitation goals explicitly targeted MAP ≥65 mmHg, (b) the administered crystalloid volume exceeded 2000 mL within the first 4 hours, or (c) more than 1500 mL of crystalloid was given before any blood product transfusion. A patient was classified into the restrictive group if (a) the documented resuscitation goals explicitly tolerated MAP 50–65 mmHg, (b) total crystalloid volume was ≤1500 mL within the first 4 hours, and (c) blood products were initiated before cumulative crystalloid exceeded 1000 mL. Patients whose resuscitation records did not clearly fit either definition were excluded from the analysis.

After initial stabilization and identification of bleeding source, all patients underwent timely hemostatic intervention (surgery, angioembolization, or thoracostomy as indicated). Subsequent fluid management after the first 24 hours was individualized and not part of the exposure definition.

(**Supplementary Table 1****).**

### 2.4 Outcome measures and detection methods

#### 2.4.1 Baseline data collection

Demographic characteristics, mechanism and nature of injury, ISS, and shock severity were recorded. Baseline disease-severity variables were also incorporated into adjusted analyses where appropriate to reduce residual confounding.

#### 2.4.2 Definitions of clinical outcomes and severity indices

Hemorrhagic shock was defined according to the American College of Surgeons Advanced Trauma Life Support (ATLS) classification-: Class I (blood loss <15%, heart rate <100, normal blood pressure), Class II (blood loss 15–30%, heart rate >100, normal blood pressure), Class III (blood loss 30–40%, heart rate >120, systolic blood pressure <90 mmHg), and Class IV (blood loss >40%, heart rate >140, systolic blood pressure <90 mmHg). For the purpose of group assignment, shock severity was categorized as mild (Class I–II) or severe (Class III–IV).

ARDS was defined according to the Berlin definition-: acute onset within 7 days of a known clinical insult, bilateral opacities on chest imaging not fully explained by effusion or collapse, respiratory failure not fully explained by cardiac failure or fluid overload, and a PaO_2_/FiO_2_ ratio ≤300 mmHg with PEEP ≥5 cm H_2_O-. Severity was graded as mild (200 < PaO_2_/FiO_2_ ≤ 300 mmHg), moderate (100 < PaO_2_/FiO_2_ ≤ 200 mmHg), or severe (PaO_2_/FiO_2_ ≤ 100 mmHg)-.

DIC was defined using the International Society on Thrombosis and Haemostasis (ISTH) overt DIC scoring system, with a score ≥5 considered diagnostic of overt DIC. The score incorporates platelet count, PT-INR, fibrinogen level, and D-dimer or fibrin degradation products.

MODS was defined as new organ dysfunction of two or more organ systems, or progressive organ dysfunction of one organ system in addition to one already dysfunctional organ system, occurring within the first 7 days after trauma. Organ dysfunction was assessed using the daily SOFA score, with dysfunction defined as a SOFA subscore ≥2 for the respective organ system.

Early resuscitation time was defined as the interval from admission (or initiation of resuscitation) to achievement of target hemodynamic stabilization, as documented in the clinical record. Target hemodynamic stabilization was defined as: (a) for the conventional group, MAP ≥65 mmHg sustained for 30 minutes; (b) for the restrictive group, MAP ≥50 mmHg with improvement in clinical signs of perfusion (improving mental status, urine output ≥0.5 mL/kg/h, or decreasing lactate) or until surgical hemostasis was achieved.

Pulmonary contusion severity was graded on admission chest CT using the Wagner classification-: Grade I (<18% air-space filling, no ventilator support required), Grade II (18–28% air-space filling, ventilator support sometimes required), and Grade III (>28% air-space filling, ventilator support always required)-.

Revised Trauma Score (RTS) was calculated using the standard formula: RTS = 0.9368 × GCS score + 0.7326 × SBP score + 0.2908 × RR score, where each variable is assigned a coded value from 0 to 4. Scores range from 0 to 7.84, with higher scores indicating better physiological status-.

Trauma Injury Severity Score (TRISS) was calculated using the RTS, ISS, and age to estimate survival probability, with separate coefficients for blunt and penetrating mechanisms-.

Massive transfusion protocol (MTP) activation was defined as transfusion of ≥10 units of packed red blood cells within 24 hours.

*Tranexamic acid (TXA) administration* was defined as a bolus dose of 1 g followed by 1 g over 8 hours, or alternative regimen per institutional protocol.

#### 2.4.3 Trauma severity indices and co-intervention variables

To characterize trauma physiology and potential confounding by concurrent interventions, we recorded the following additional variables from the medical records and trauma registry.

##### 2.4.3.1 Trauma severity indices

The Revised Trauma Score (RTS) was calculated from Glasgow Coma Scale, systolic blood pressure, and respiratory rate at admission using the standard formula: RTS = 0.9368×GCS + 0.7326×SBP + 0.2908×RR, with values ranging from 0 to 7.84 (higher indicates better physiology) (Champion et al., 1989). The Trauma Injury Severity Score (TRISS) was calculated using the RTS, ISS, and age to estimate survival probability (Boyd et al., 1987). Chest Abbreviated Injury Scale (AIS) was determined from the trauma registry, with scores ranging from 1 (minor) to 6 (maximal/unsurvivable). Pulmonary contusion severity was graded on admission chest CT using a standardized scale (grades 1–4: grade 1 = <10% lung volume; grade 2 = 10–25%; grade 3 = 25–50%; grade 4 = >50%) (Wagner et al., 1988); grades 3–4 were considered severe. Bilateral lung involvement was defined as pulmonary contusions present in both hemithoraces on admission CT. PaO_2_/FiO_2_ ratio was calculated from the first arterial blood gas obtained within 1 hour of admission. Base deficit trajectory was defined as the worst (most negative) base deficit value recorded within the first 6 hours of admission, reflecting cumulative metabolic acidosis burden.

##### 2.4.3.2 Co-intervention data

To assess potential confounding by concurrent interventions, we recorded MTP activation(≥10 units RBC within 24 h), TXA administration (1 g bolus + 1 g over 8 h), 24-h FFP: RBC and PLT: RBC ratios, lowest fibrinogen level within 6 h, and cryoprecipitate use (binary). These variables were balanced between groups after matching (**Table 1B**) and were included in multivariable sensitivity analyses to evaluate their confounding effects.

**Table 1A:** Baseline characteristics of the original cohort before propensity score matching.

Characteristic	Conventional (n = 143)	Restrictive (n = 143)	SMD	P-value
Age (years), mean ± SD	44.8 ± 11.2	46.1 ± 10.9	0.12	0.312
Male, n (%)	96 (67.1)	91 (63.6)	0.07	0.534
ISS score, mean ± SD	27.4 ± 6.8	28.9 ± 6.5	0.22	0.048
RTS, mean ± SD	6.8 ± 1.2	6.4 ± 1.3	0.32	0.006
TRISS, mean ± SD	0.72 ± 0.18	0.66 ± 0.19	0.33	0.005
Shock severity, n (%)			0.18	0.124
- Mild	41 (28.7)	32 (22.4)		
- Moderate	62 (43.4)	61 (42.7)		
- Severe	40 (28.0)	50 (35.0)		
Mechanism of injury, n (%)			0.14	0.267
- Traffic accident	71 (49.7)	68 (47.6)		
- Fall	38 (26.6)	41 (28.7)		
- Crush	22 (15.4)	24 (16.8)		
- Other	12 (8.4)	10 (7.0)		
Chest AIS, mean ± SD	3.6 ± 0.8	3.9 ± 0.9	0.35	0.003
Pulmonary contusion severity (CT grade 3–4), n (%)	34 (23.8)	45 (31.5)	0.17	0.142
Bilateral lung involvement, n (%)	28 (19.6)	36 (25.2)	0.14	0.258
Need for mechanical ventilation, n (%)	52 (36.4)	61 (42.7)	0.13	0.278
PaO_2_/FiO_2_ ratio, mean ± SD	285.4 ± 72.3	268.7 ± 78.4	0.23	0.054
Respiratory rate (breaths/min), mean ± SD	26.4 ± 6.2	28.7 ± 6.8	0.35	0.003
Body temperature (°C), mean ± SD	36.2 ± 0.7	35.9 ± 0.8	0.4	0.001
pH, mean ± SD	7.28 ± 0.12	7.24 ± 0.13	0.32	0.007
Ionized calcium (mmol/L), mean ± SD	1.08 ± 0.14	1.04 ± 0.15	0.28	0.019
Base deficit trajectory (worst 6 h, mEq/L), mean ± SD	-7.2 ± 2.6	-8.5 ± 2.9	0.47	<0.001
Admission lactate (mmol/L), mean ± SD	4.6 ± 1.7	5.1 ± 1.8	0.28	0.016
Admission base deficit (mEq/L), mean ± SD	-6.5 ± 2.5	-7.3 ± 2.6	0.31	0.008
Time from injury to admission (h), mean ± SD	2.7 ± 1.5	3.0 ± 1.6	0.19	0.087
GCS score, mean ± SD	13.2 ± 2.4	12.8 ± 2.6	0.16	0.152
Admission systolic blood pressure (mmHg), mean ± SD	84.6 ± 15.3	81.2 ± 16.7	0.21	0.064
Admission heart rate (beats/min), mean ± SD	118.4 ± 22.7	122.6 ± 24.1	0.18	0.103
Hemoglobin (g/L), mean ± SD	102.3 ± 18.6	98.7 ± 19.4	0.19	0.092
PT (s), mean ± SD	16.2 ± 2.8	17.1 ± 3.2	0.3	0.011
APTT (s), mean ± SD	41.5 ± 6.2	43.8 ± 6.9	0.35	0.003
Fibrinogen (g/L), mean ± SD	2.1 ± 0.7	1.8 ± 0.6	0.46	**<0.001**
Mechanical ventilation at admission, n (%)	52 (36.4)	61 (42.7)	0.13	0.278
Chest tube drainage performed, n (%)	98 (68.5)	103 (72.0)	0.08	0.521
Associated abdominal injury, n (%)	31 (21.7)	35 (24.5)	0.07	0.578
Associated pelvic injury, n (%)	18 (12.6)	22 (15.4)	0.08	0.498
Pulmonary contusion, n (%)	87 (60.8)	94 (65.7)	0.1	0.392
Flail chest, n (%)	42 (29.4)	48 (33.6)	0.09	0.448
Massive hemothorax, n (%)	53 (37.1)	59 (41.3)	0.09	0.468
Time to hemostatic intervention (min), mean ± SD	72.5 ± 28.6	63.8 ± 24.1	0.33	0.005
Bleeding control modality, n (%)			0.21	0.038
– Thoracotomy/surgery	92 (64.3)	98 (68.5)		
– Angioembolization	22 (15.4)	26 (18.2)		
– Conservative (chest tube only)	29 (20.3)	19 (13.3)		
RBC transfusion (units, 24 h), median (IQR)	3.0 (1–5)	2.0 (0–4)	0.35	0.008
FFP transfusion (units, 24 h), median (IQR)	2.0 (0–4)	1.0 (0–3)	0.28	0.017
Platelet transfusion (units, 24 h), median (IQR)	0 (0–2)	0 (0–1)	0.22	0.043
MTP activation, n (%)	25 (17.5)	14 (9.8)	0.23	0.056
TXA administered, n (%)	52 (36.4)	57 (39.9)	0.07	0.544
FFP: RBC ratio (24 h), median (IQR)	0.50 (0.33–0.75)	0.60 (0.40–0.83)	0.25	0.033
PLT: RBC ratio (24 h), median (IQR)	0.08 (0–0.20)	0.10 (0–0.25)	0.15	0.142

Values are mean ± SD, n (%), or median (IQR). SMD, standardized mean difference; AIS, Abbreviated Injury Scale; RTS, Revised Trauma Score; TRISS, Trauma Injury Severity Score; MTP, massive transfusion protocol; TXA, tranexamic acid; FFP, fresh frozen plasma; PLT, platelets; RBC, red blood cells. P-values: t-test or Mann-Whitney U test for continuous variables; χ² or Fisher’s exact test for categorical variables. SMD <0.1 indicates adequate balance. Bold values indicate newly added variables.

**Table 1B:** Baseline characteristics of the matched cohort after propensity score matching.

Characteristic	Conventional (n = 100)	Restrictive (n = 100)	SMD	P-value
Age (years), mean ± SD	45.2 ± 10.8	45.5 ± 11.2	0.03	0.845
Male, n (%)	67 (67.0)	65 (65.0)	0.04	0.762
ISS score, mean ± SD	28.1 ± 6.3	27.9 ± 6.1	0.03	0.819
RTS, mean ± SD	6.6 ± 1.2	6.7 ± 1.1	0.08	0.546
TRISS, mean ± SD	0.69 ± 0.17	0.70 ± 0.16	0.06	0.667
Shock severity, n (%)			0.06	0.743
- Mild	25 (25.0)	24 (24.0)		
- Moderate	43 (43.0)	42 (42.0)		
- Severe	32 (32.0)	34 (34.0)		
Mechanism of injury, n (%)			0.08	0.582
- Traffic accident	51 (51.0)	48 (48.0)		
- Fall	27 (27.0)	29 (29.0)		
- Crush	15 (15.0)	16 (16.0)		
- Other	7 (7.0)	7 (7.0)		
Chest AIS, mean ± SD	3.7 ± 0.8	3.8 ± 0.8	0.09	0.378
Pulmonary contusion severity (CT grade 3–4), n (%)	28 (28.0)	30 (30.0)	0.04	0.754
Bilateral lung involvement, n (%)	22 (22.0)	24 (24.0)	0.05	0.734
Need for mechanical ventilation, n (%)	40 (40.0)	42 (42.0)	0.04	0.771
PaO_2_/FiO_2_ ratio, mean ± SD	276.3 ± 74.2	278.4 ± 72.8	0.03	0.838
Respiratory rate (breaths/min), mean ± SD	27.2 ± 6.4	26.8 ± 6.2	0.06	0.653
Body temperature (°C), mean ± SD	36.0 ± 0.7	36.1 ± 0.7	0.09	0.312
pH, mean ± SD	7.26 ± 0.12	7.27 ± 0.11	0.09	0.546
Ionized calcium (mmol/L), mean ± SD	1.05 ± 0.14	1.06 ± 0.13	0.07	0.602
Base deficit trajectory (worst 6 h, mEq/L), mean ± SD	-7.8 ± 2.7	-7.5 ± 2.6	0.09	0.428
Admission lactate (mmol/L), mean ± SD	4.8 ± 1.6	4.9 ± 1.7	0.06	0.667
Admission base deficit (mEq/L), mean ± SD	-6.8 ± 2.3	-7.0 ± 2.4	0.08	0.547
Time from injury to admission (h), mean ± SD	2.8 ± 1.4	2.9 ± 1.5	0.07	0.623
GCS score, mean ± SD	13.0 ± 2.5	12.9 ± 2.6	0.04	0.781
Admission systolic blood pressure (mmHg), mean ± SD	83.2 ± 16.0	82.5 ± 16.5	0.04	0.758
Admission heart rate (beats/min), mean ± SD	120.1 ± 23.4	121.3 ± 24.0	0.05	0.714
Hemoglobin (g/L), mean ± SD	100.5 ± 19.0	99.8 ± 19.5	0.04	0.792
PT (s), mean ± SD	16.5 ± 2.9	16.7 ± 3.0	0.07	0.632
APTT (s), mean ± SD	42.2 ± 6.3	42.8 ± 6.5	0.09	0.508
**Fibrinogen (g/L), mean ± SD**	**1.9 ± 0.6**	**2.0 ± 0.7**	**0.09**	**0.286**
Mechanical ventilation at admission, n (%)	40 (40.0)	42 (42.0)	0.04	0.771
Chest tube drainage performed, n (%)	70 (70.0)	72 (72.0)	0.04	0.756
Associated abdominal injury, n (%)	22 (22.0)	24 (24.0)	0.05	0.734
Associated pelvic injury, n (%)	15 (15.0)	16 (16.0)	0.03	0.844
Pulmonary contusion, n (%)	62 (62.0)	64 (64.0)	0.04	0.768
Flail chest, n (%)	30 (30.0)	32 (32.0)	0.04	0.758
Massive hemothorax, n (%)	38 (38.0)	40 (40.0)	0.04	0.771
Time to hemostatic intervention (min), mean ± SD	67.2 ± 25.1	64.8 ± 24.5	0.1	0.486
Bleeding control modality, n (%)			0.07	0.758
– Thoracotomy/surgery	69 (69.0)	67 (67.0)		
– Angioembolization	17 (17.0)	18 (18.0)		
– Conservative (chest tube only)	14 (14.0)	15 (15.0)		
RBC transfusion (units, 24 h), median (IQR)	2.0 (0–4)	2.0 (0–3)	0.09	0.412
FFP transfusion (units, 24 h), median (IQR)	1.0 (0–3)	1.0 (0–2)	0.08	0.523
Platelet transfusion (units, 24 h), median (IQR)	0 (0–1)	0 (0–1)	0.05	0.697
MTP activation, n (%)	8 (8.0)	6 (6.0)	0.08	0.580
TXA administered, n (%)	42 (42.0)	45 (45.0)	0.06	0.666
FFP:RBC ratio (24 h), median (IQR)	0.56 (0.33–0.80)	0.60 (0.40–0.83)	0.09	0.327
PLT:RBC ratio (24 h), median (IQR)	0.08 (0–0.25)	0.10 (0–0.29)	0.07	0.458

Values are mean ± SD, n (%), or median (IQR). SMD, standardized mean difference; AIS, Abbreviated Injury Scale; RTS, Revised Trauma Score; TRISS, Trauma Injury Severity Score; MTP, massive transfusion protocol; TXA, tranexamic acid; FFP, fresh frozen plasma; PLT, platelets; RBC, red blood cells. P-values: t-test or Mann-Whitney U test for continuous variables; χ² or Fisher's exact test for categorical variables. SMD <0.1 indicates adequate balance. Bold values indicate newly added variables.

#### 2.4.4 Fluid resuscitation-related indicators

Fluid-related variables included total early fluid input, early resuscitation time, colloid use, cumulative fluid balance at 24, 48, and 72 h, and Fluid Efficiency Ratio (FER). Total early fluid input was defined as the sum of all intravenous fluids administered during the early resuscitation phase (first 4 hours), including crystalloids (lactated Ringer’s or normal saline), colloids (hydroxyethyl starch or albumin), and blood products (packed red blood cells, fresh frozen plasma, and platelets). Early resuscitation time was defined as the interval from admission to achievement of target hemodynamic stabilization as documented in the clinical record. Colloid usage referred to the total volume of colloids administered during the early resuscitation phase. Cumulative fluid balance was calculated at 24 h, 48 h, and 72 h as total intake minus total output (mL). FER was defined as the increase in mean arterial pressure (MAP) per liter of fluid administered during the early resuscitation phase (mmHg/L). This exploratory metric was intended to reflect hemodynamic responsiveness to fluid administration; it assumes that MAP is a valid surrogate for hemodynamic efficacy and that the relationship between fluid volume and MAP response is approximately linear—assumptions that have not been validated.

#### 2.4.5 Novel composite metrics (exploratory)

To summarize multi-time-point physiological trajectories, we developed three composite indices *post hoc* for hypothesis-generating purposes. These are not validated clinical tools and should be interpreted only as exploratory descriptors. No adjustments for multiple comparisons were applied.

##### 2.4.5.1 Oxygen debt index

The ODI was defined as the time-integrated cumulative oxygen delivery (DO_2_) deficit over the first 72 hours. For each patient, we calculated the hourly area under the curve of DO_2_ deficit relative to an age-predicted normal DO_2_ value (600 mL/min for adults aged 18–40 years, with a reduction of 5 mL/min per decade thereafter (Ebihara et al., 2022)), using the formula:

ODI = Σ_t_=_0_^7^² max(0, DO_2__normal − DO_2__observed(t)) × Δt

where Δt = 1 hour. Missing DO_2_ values (<5%) were imputed by linear interpolation; patients with >20% missing data (n=6) were excluded. *Units: mL/min × hours.*

##### 2.4.5.2 Coagulation recovery rate

The CRR was defined as the percentage improvement in activated partial thromboplastin time (APTT) from baseline:

CRR(t) = [(APTT_baseline − APTT(t))/APTT_baseline] × 100%

This metric assumes APTT is a sufficient linear measure of coagulation recovery—an assumption not accounting for platelet function, fibrinogen, or other factors, and not validated against viscoelastic assays. For patients with missing baseline APTT (n=4), the admission value was used; the 72-h CRR served as the primary summary measure. *Units: %.*

##### 2.4.5.3 Inflammation burden score

The IBS was derived from principal component analysis (PCA) of IL-6, IL-10, and TNF-α measured at 0, 6, 12, 24, 48, and 72 h. PCA was performed on log-transformed values; the first principal component (PC1) accounted for 68.4% of total variance, with loadings of 0.62 (IL-6), 0.58 (TNF-α), and −0.53 (IL-10). IBS was defined as the PC1 score standardized to mean 0 and SD 1 in the overall cohort. Internal consistency (Cronbach’s α) was 0.79 (95% CI 0.74–0.84) across all time points. *Units: SD score.*

All three indices are hypothesis-generating descriptors and require external validation before clinical use.

#### 2.4.6 Physiological and laboratory measurements

Peripheral venous blood samples were collected before resuscitation and at 6, 12, 24, 48, and 72 h. The following parameters were measured at each time point: (1) oxygen metabolism—SvO_2_, DO_2_, VO_2_, and arterial lactate, with lactate clearance calculated as [(baseline lactate − lactate at time t)/baseline lactate] × 100%; (2) coagulation function—APTT, PT, and HCT, with the APTT/PT ratio recorded as a supplementary descriptor; and (3) inflammatory markers—IL-6, IL-10, and TNF-α via standardized immunoassays. The three exploratory composite indices (ODI, CRR, and IBS) were additionally calculated as defined in the “Novel composite metrics” section above.

#### 2.4.7 Clinical outcomes

Clinical outcomes included 28-day survival and major in-hospital complications during the early treatment course, particularly acute respiratory distress syndrome (ARDS), disseminated intravascular coagulation (DIC), and multiple organ dysfunction syndrome (MODS). A composite adverse outcome was also analyzed to reflect early clinically relevant deterioration.

Daily SOFA scores from admission through day 7 were collected, and the area under the SOFA curve (AUC-SOFA) was calculated to reflect cumulative organ dysfunction burden.

Ventilator-free days (VFDs) and ICU-free days (IFDs) within 28 days were also recorded; both were assigned as 0 in patients who died before day 28.

### 2.5 Statistical analysis

Analyses were performed using SPSS version 22.0 and R version 4.2.1. Continuous variables were summarized as mean ± standard deviation or median (interquartile range), as appropriate, and categorical variables as number (percentage). Between-group comparisons used the independent-samples t-test or Mann–Whitney U test for continuous variables and the χ² test or Fisher’s exact test for categorical variables.

To minimize selection bias, PSM was performed using a logistic regression model with treatment assignment as the dependent variable and age, sex, Injury Severity Score (ISS), and shock severity as covariates. The model showed acceptable discrimination (C-statistic = 0.71, 95% CI 0.65–0.77) and calibration (Hosmer–Lemeshow test: χ² = 8.3, df = 8, P = 0.40). Scores were trimmed at the 1st and 99th percentiles (n = 6 excluded), and 1:1 nearest-neighbor matching without replacement was performed with a caliper of 0.2 standard deviations of the logit. Balance after matching was assessed using standardized mean differences (SMDs), with SMD < 0.1 considered well-balanced; all SMDs were ≤0.09. Alternative matching specifications (1:2 matching, caliper 0.1, and matching with replacement) yielded similar results.

We did not include RTS, TRISS, or detailed thoracic injury variables (Chest AIS, PaO_2_/FiO_2_ ratio, fibrinogen, base deficit) in the matching model by design. These variables represent early physiological responses to injury and may lie on the causal pathway between the resuscitation strategy and the outcomes. Conditioning on post-injury, pre-resuscitation variables that could be affected by the strategy itself can introduce overadjustment bias. Therefore, these variables were purposefully reserved for sensitivity analyses as prespecified covariates in multivariable models to assess the robustness of the primary findings rather than being used as matching variables.

Serial variables (oxygen metabolism, coagulation, inflammatory markers, and SOFA scores) were analyzed using linear mixed-effects models with restricted maximum likelihood estimation, which better handles missing data and unequal follow-up than repeated-measures ANOVA. Models included fixed effects for time (6 time points: baseline, 6, 12, 24, 48, 72 h), group, and group-by-time interaction, with a random intercept for each patient and an unstructured covariance matrix. Kenward–Roger degrees of freedom were applied for small-sample inference. Model assumptions were verified visually using Q-Q and residual-vs-fitted plots, with no major violations detected. Between-group comparisons at individual time points used model-estimated marginal means with Bonferroni correction.

The overall proportion of missing values was 7.4% (range 0–14.2%), with missingness not significantly associated with resuscitation strategy (P = 0.32) or 28-day mortality (P = 0.47), supporting a missing-at-random assumption. For continuous serial variables, missing values were imputed using linear interpolation when at least one adjacent measurement was available; values after death were not imputed. Patients with >20% missing data for a given variable (n = 12, 6%) were excluded from analyses involving that variable. Complete-case and multiple imputation sensitivity analyses yielded results consistent with the primary analysis. To further quantify the potential impact of unmeasured confounding on the observed 28-day survival benefit, we calculated the E-value for the primary Cox regression result (HR = 0.23). The E-value represents the minimum strength of association that an unmeasured confounder would need to have with both the treatment and the outcome to fully explain away the observed association. A large E-value would suggest that the finding is robust to unmeasured confounding.

To assess robustness to missing data assumptions and survivor bias, we performed: (1) complete-case analysis, (2) worst-case imputation (assigning the worst observed value within each group to patients who died before 72 h), and (3) multiple imputation using chained equations. To specifically address survivor bias in the serial physiological data (0–72 h), a prespecified sensitivity analysis was conducted restricted to patients who survived to 72 hours. This ‘survivors-only’ analysis aimed to determine whether the observed physiological benefits of the restrictive strategy persisted when comparing only patients who were alive at the final measurement time point. To assess confounding by trauma severity and concurrent interventions, additional models adjusted for RTS, TRISS, Chest AIS, PaO_2_/FiO_2_ ratio, MTP activation, TXA use, FFP: RBC ratio, PLT: RBC ratio, fibrinogen level, and cryoprecipitate use. Additional robustness checks included inverse probability weighting, exclusion of extreme fluid volumes (>4000 mL), and adjustment for mechanism of injury and time to admission. All sensitivity analyses yielded effect estimates consistent with the primary findings.

Kaplan–Meier analysis with the log-rank test was used for 28-day survival. Multivariable Cox regression was performed for 28-day mortality, and multivariable logistic regression for the composite adverse outcome; covariates were prespecified based on clinical plausibility and baseline severity.

Principal component analysis derived the exploratory IBS from IL-6, IL-10, and TNF-α; hierarchical clustering was applied to describe inflammatory phenotypes, both interpreted as hypothesis-generating. Subgroup analyses (by age, ISS, shock severity, baseline lactate, and admission SOFA) were exploratory, with emphasis on directional consistency. Spearman correlation examined associations between total fluid volume and post-resuscitation variables, with partial correlation adjusting for baseline severity as a robustness check. Mediation analysis exploring 24-h lactate clearance and 24-h IL-6 as potential intermediaries was prespecified as exploratory only, without causal interpretation. All tests were two-sided, with P < 0.05 considered statistically significant.

## 3 Results

### 3.1 Study population and baseline characteristics

**Figure 1:**
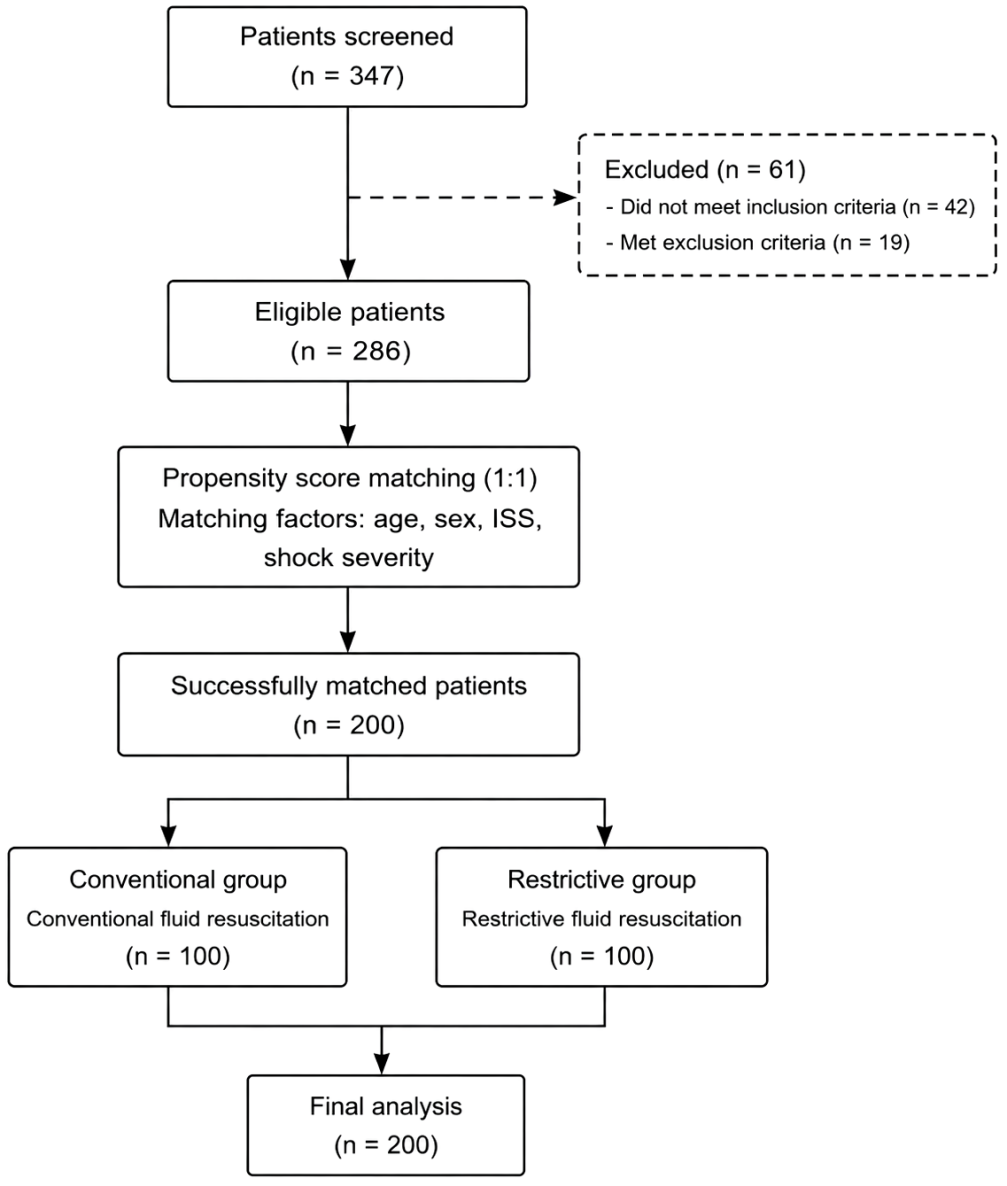
Study flow diagram.

Between June 2023 and December 2025, 347 patients with severe chest trauma and hemorrhagic shock were screened; 286 met eligibility criteria and constituted the original cohort. After propensity score matching, 100 matched pairs (200 patients) were included as the primary analytic cohort (**Figure 1**).

**Figure 2:**
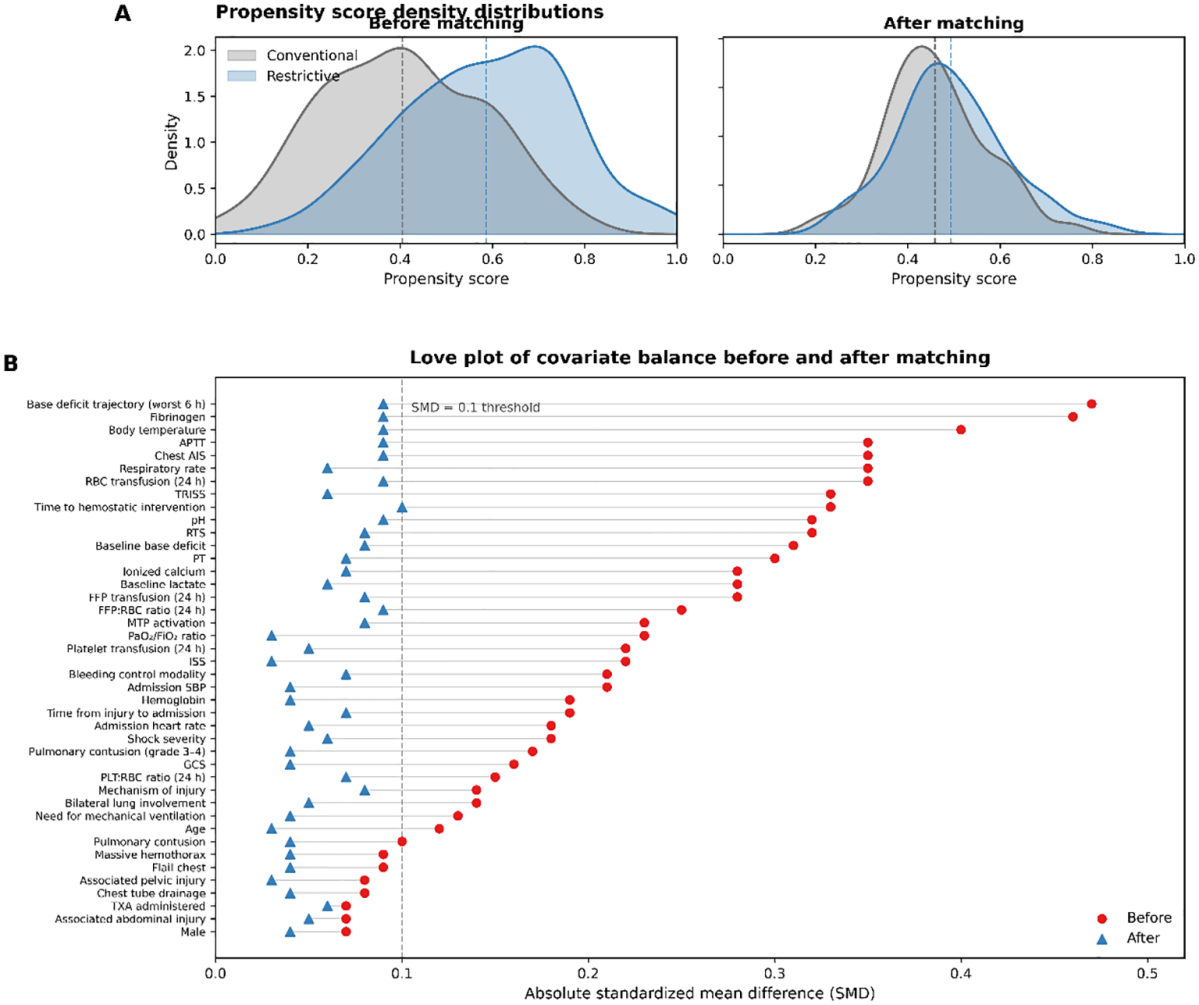
Covariate balance before and after propensity score matching. **(A)** Propensity score density plots before (left) and after (right) matching. **(B)** Standardized mean differences (SMD) of covariates before and after matching.

Before matching, propensity score distributions differed substantially between groups (**Figure 2A**, left panel). After matching, distributions overlapped nearly completely (**Figure 2A**, right panel), and all absolute SMDs were reduced to below 0.1 (**Figure 2B**), indicating adequate covariate balance. Baseline characteristics of the original andmatched cohorts are shown in **Tables 1A, B**, respectively. In the matched cohort, all SMDs were ≤0.09, confirming successful balance between groups.

Missing data for serial measurements were infrequent (overall 7.4%), with no significant association with resuscitation strategy or mortality (both P > 0.30). Linear interpolation was used for imputation, and complete-case sensitivity analyses confirmed the robustness of the findings.

### 3.2 Fluid exposure and early resuscitation efficiency

**Figure 3:**
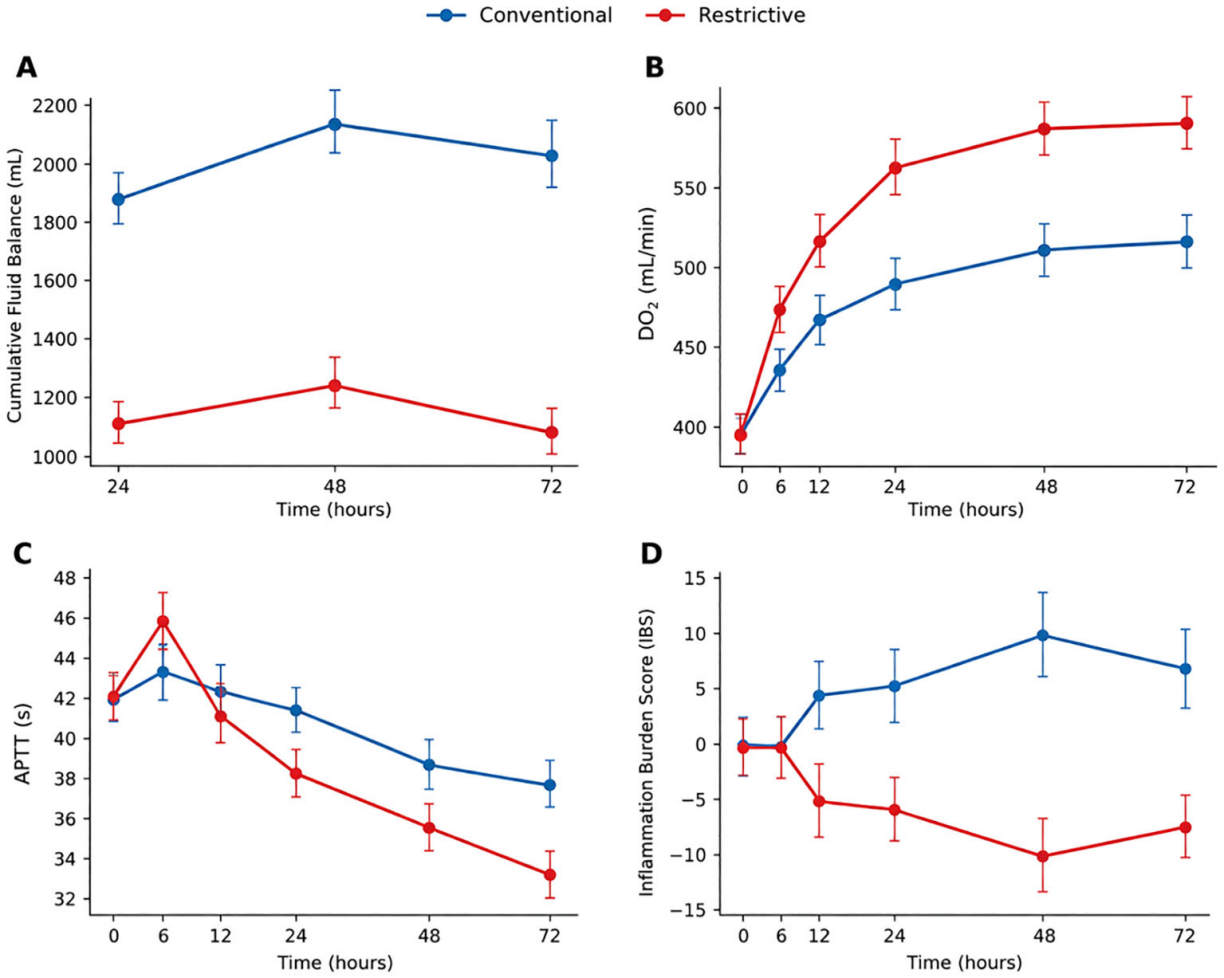
Serial physiologic trajectories over the first 72 h. **(A)** Cumulative fluid balance. **(B)** Oxygen delivery (DO_2_). **(C)** Activated partial thromboplastin time (APTT). **(D)** Inflammation Burden Score (IBS) derived from principal component analysis of IL-6, IL-10, and TNF-α. Data are presented as mean ± SEM; between-group comparisons at each time point were interpreted in the context of repeated-measures analysis.

After matching, the restrictive group received significantly less total fluid than the conventional group (1984.6 ± 325.4 mL vs. 2845.7 ± 492.3 mL, P < 0.001; **Table 2**), with no significant difference in early resuscitation time or colloid usage, indicating that lower fluid exposure was not achieved by delaying care. FER was significantly higher in the restrictive group (13.2 ± 3.4 vs. 8.5 ± 2.7 mmHg/L, P < 0.001), suggesting greater hemodynamic efficiency per unit fluid administered. Cumulative fluid balance remained consistently lower in the restrictive group over the first 72 h, with a significant group-by-time interaction and widening between-group differences over time (**Figure 3A**).

**Table 2:** Fluid resuscitation parameters.

Parameter	Conventional (n = 100)	Restrictive (n = 100)	Mean Difference (95% CI)	P-value
Total fluid input (mL)	2845.7 ± 492.3	1984.6 ± 325.4	−861.1 (−982.4 to −739.8)	<0.001
Early resuscitation time (min)	24.1 ± 7.8	22.6 ± 6.9	−1.5 (−3.6 to 0.6)	0.156
Colloid usage (mL)	752.3 ± 238.5	793.6 ± 281.4	41.3 (−31.2 to 113.8)	0.263
Fluid Efficiency Ratio (mmHg/L)	8.5 ± 2.7	13.2 ± 3.4	4.7 (3.8–5.6)	<0.001
24 h cumulative balance (mL)	1892.4 ± 412.7	1123.8 ± 287.6	−768.6 (−868.4 to −668.8)	<0.001
48 h cumulative balance (mL)	2135.6 ± 467.3	1247.2 ± 312.5	−888.4 (−1001.2 to −775.6)	<0.001
72 h cumulative balance (mL)	2018.9 ± 438.2	1086.4 ± 276.8	−932.5 (−1036.7 to −828.3)	<0.001

Values are mean ± SD. FER, Fluid Efficiency Ratio (mmHg/L). P-values from independent-samples t-test. Total fluid input includes crystalloids, colloids, and blood products.

### 3.3 Exploratory composite indices

The exploratory composite indices—ODI, CRR, and IBS—all demonstrated consistent between-group differences favoring the restrictive strategy. The restrictive group had significantly lower ODI (124.7 ± 38.2 vs. 187.3 ± 45.6, mean difference −62.6, 95% CI −74.1 to −51.1, P < 0.001), higher CRR at 72 h (56.8% ± 11.4% vs. 38.2% ± 10.1%, P < 0.001), and lower AUC-IBS over 72 h (56.4 ± 18.2 vs. 89.7 ± 24.6, P < 0.001). CRR showed a strong negative correlation with total fluid volume (Spearman’s ρ = −0.52, P < 0.001). The IBS trajectory declined more steeply in the restrictive group from 12 h onward (P for group-by-time interaction < 0.001), with Cronbach’s α of 0.79 across all time points indicating acceptable internal consistency. As these are post-hoc, exploratory metrics derived from serial physiological data, they should be interpreted as hypothesis-generating descriptors rather than validated clinical endpoints, and their properties require confirmation in independent prospective cohorts.

### 3.4 Physiological trajectories

#### 3.4.1 Oxygen metabolism

Both groups showed progressive improvement in SvO_2_, VO_2_, and DO_2_ over time, but the restrictive group demonstrated earlier and more pronounced recovery, with significant time, group, and group-by-time interaction effects (**Figure 3B**; **Table 3**). Lactate clearance was consistently higher in the restrictive group at all time points. These patterns suggest that lower fluid exposure was not associated with compromised oxygen delivery; rather, oxygen-transport recovery appeared more favorable in the restrictive group, consistent with the lower ODI reported above.

**Table 3:** Oxygen metabolism indicators at key time points.

Parameter	Group	Baseline	6 h	12 h	24 h	48 h	72 h	P-time	P-group	P-interaction
SvO_2_ (%)	Conventional	58.3 ± 5.2	62.4 ± 5.8	65.7 ± 5.4	68.2 ± 4.9	69.5 ± 4.6	70.1 ± 4.3	<0.001	0.002	0.008
	Restrictive	57.9 ± 5.4	65.2 ± 5.9*	69.3 ± 5.1*	72.4 ± 4.7*	73.8 ± 4.4*	74.5 ± 4.2*			
VO_2_ (mL/min)	Conventional	184.3 ± 28.7	198.6 ± 30.2	212.4 ± 28.9	223.7 ± 27.3	228.4 ± 26.8	231.2 ± 25.9	<0.001	0.004	0.015
	Restrictive	182.7 ± 29.1	207.3 ± 31.4*	226.8 ± 29.7*	241.5 ± 28.2*	248.3 ± 27.1*	252.6 ± 26.3*			
DO_2_ (mL/min)	Conventional	382.4 ± 62.3	432.7 ± 68.4	468.3 ± 65.2	492.6 ± 61.8	508.4 ± 60.3	514.7 ± 59.2	<0.001	<0.001	<0.001
	Restrictive	378.6 ± 63.7	467.2 ± 71.5*	518.6 ± 67.8*	556.3 ± 64.2*	582.7 ± 62.5*	594.2 ± 61.4*			
Oxygen Debt Index	Conventional	–	–	–	–	–	187.3 ± 45.6	–	–	–
	Restrictive	–	–	–	–	–	124.7 ± 38.2*			
Lactate clearance (%)	Conventional	–	21.8 ± 8.4	32.6 ± 9.1	44.3 ± 10.5	53.7 ± 11.4	–	–	–	–
	Restrictive	–	32.4 ± 9.7*	45.2 ± 10.3*	58.6 ± 11.2*	67.3 ± 10.8*	–			

Values are mean ± SD. ODI, Oxygen Debt Index (mL/min × hours). P-time, P-group, P-interaction from linear mixed-effects models. *P < 0.05 for between-group comparison at that time point (Bonferroni-corrected). ODI is exploratory and not a validated clinical tool.

Detailed serial values are shown in **Table 3**.

#### 3.4.2 Coagulation function

APTT, PT, and HCT improved over time in both groups, but recovery was faster in the restrictive group, with significant group-by-time interactions (**Figure 3C**; **Table 4**). The APTT/PT ratio normalized more rapidly in the restrictive group, consistent with the higher CRR reported above. These findings align with less persistent hemodilution and a more favorable early hemostatic milieu under the restrictive strategy.

**Table 4:** Coagulation function indicators over time.

Parameter	Group	Baseline	6 h	12 h	24 h	48 h	72 h	P-time	P-group	P-interaction
APTT (s)	Conventional	42.7 ± 5.8	44.3 ± 6.2	43.1 ± 5.9	40.8 ± 5.4	38.6 ± 5.1	36.9 ± 4.8	<0.001	0.003	0.007
	Restrictive	43.1 ± 6.0	45.2 ± 6.4	41.6 ± 5.7	37.4 ± 5.0*	34.8 ± 4.7*	32.5 ± 4.3*			
PT (s)	Conventional	16.8 ± 2.4	17.5 ± 2.6	16.9 ± 2.4	16.1 ± 2.2	15.4 ± 2.0	14.8 ± 1.9	<0.001	0.008	0.013
	Restrictive	17.0 ± 2.5	17.8 ± 2.7	16.3 ± 2.3	15.2 ± 2.1*	14.3 ± 1.9*	13.6 ± 1.7*			
HCT (%)	Conventional	28.4 ± 4.2	26.1 ± 3.9	27.2 ± 4.0	28.9 ± 4.1	30.3 ± 4.3	31.5 ± 4.4	<0.001	<0.001	<0.001
	Restrictive	28.1 ± 4.3	25.8 ± 4.0	28.4 ± 4.2	31.2 ± 4.4*	33.8 ± 4.6*	36.3 ± 4.8*			
APTT/PT ratio	Conventional	2.54 ± 0.32	2.53 ± 0.34	2.55 ± 0.33	2.53 ± 0.31	2.51 ± 0.30	2.49 ± 0.29	0.342	0.045	0.078
	Restrictive	2.53 ± 0.33	2.54 ± 0.35	2.55 ± 0.34	2.46 ± 0.30	2.43 ± 0.29	2.39 ± 0.28*			
Coagulation Recovery Rate (%)	Conventional	–	–	–	24.7 ± 8.3	31.5 ± 9.2	38.2 ± 10.1	–	–	–
	Restrictive	–	–	–	38.4 ± 9.5*	47.3 ± 10.6*	56.8 ± 11.4*			

Values are mean ± SD. CRR, Coagulation Recovery Rate (%). P-time, P-group, P-interaction from linear mixed-effects models. *P < 0.05 for between-group comparison at that time point (Bonferroni-corrected). CRR is exploratory and not a validated clinical tool.

#### 3.4.3 Inflammatory response

All three cytokines decreased over time in both groups, but declines were steeper in the restrictive group, with significant group and interaction effects (**Figure 3D**; **Table 5**). The IL-6/IL-10 ratio declined more rapidly in the restrictive group, suggesting earlier rebalancing between pro-inflammatory and counter-regulatory responses. Hierarchical clustering further indicated that restrictive resuscitation was associated with a higher proportion of patients showing a “rapid-resolver” phenotype and a lower proportion with persistent inflammatory activation. These findings, together with the lower IBS reported above, support a biologically plausible association between lower early fluid burden and attenuated inflammatory activation in severe chest trauma.

**Table 5:** Inflammatory markers and composite indices.

Parameter	Group	Baseline	6 h	12 h	24 h	48 h	72 h	P-time	P-group	P-interaction
IL-6 (pg/mL)	Conventional	86.4 ± 24.7	94.2 ± 26.8	82.7 ± 23.5	67.3 ± 20.4	52.8 ± 17.6	41.2 ± 14.3	<0.001	<0.001	<0.001
	Restrictive	88.1 ± 25.3	92.6 ± 25.9	74.5 ± 21.6*	54.7 ± 18.2*	38.4 ± 14.5*	27.6 ± 11.2*			
IL-10 (pg/mL)	Conventional	32.7 ± 10.4	36.8 ± 11.5	33.4 ± 10.2	28.6 ± 9.4	24.3 ± 8.2	20.7 ± 7.3	<0.001	0.002	0.009
	Restrictive	33.4 ± 10.8	37.2 ± 11.8	30.7 ± 9.8	24.2 ± 8.5*	19.5 ± 7.4*	15.8 ± 6.2*			
TNF-α (pg/mL)	Conventional	42.6 ± 13.8	45.3 ± 14.5	40.8 ± 13.2	35.4 ± 11.9	30.7 ± 10.4	26.3 ± 9.1	<0.001	0.004	0.011
	Restrictive	43.2 ± 14.1	44.8 ± 14.2	37.6 ± 12.5	30.2 ± 10.8*	24.5 ± 9.3*	19.8 ± 7.8*			
Inflammation Burden Score	Conventional	1.82 ± 0.54	1.94 ± 0.58	1.68 ± 0.51	1.36 ± 0.44	1.08 ± 0.37	0.86 ± 0.31	<0.001	<0.001	<0.001
	Restrictive	1.85 ± 0.56	1.91 ± 0.57	1.52 ± 0.48*	1.12 ± 0.39*	0.76 ± 0.29*	0.52 ± 0.23*			
IL-6/IL-10 ratio	Conventional	2.64 ± 0.82	2.56 ± 0.79	2.48 ± 0.76	2.35 ± 0.71	2.17 ± 0.65	1.99 ± 0.58	<0.001	0.007	0.023
	Restrictive	2.63 ± 0.83	2.49 ± 0.77	2.43 ± 0.74	2.26 ± 0.68	1.97 ± 0.59*	1.75 ± 0.51*			

Values are mean ± SD. IBS, Inflammation Burden Score (SD score). P-time, P-group, P-interaction from linear mixed-effects models. *P < 0.05 for between-group comparison at that time point (Bonferroni-corrected). IBS is exploratory and not a validated clinical tool.

### 3.5 Clinical outcomes and organ function

**Figure 4:**
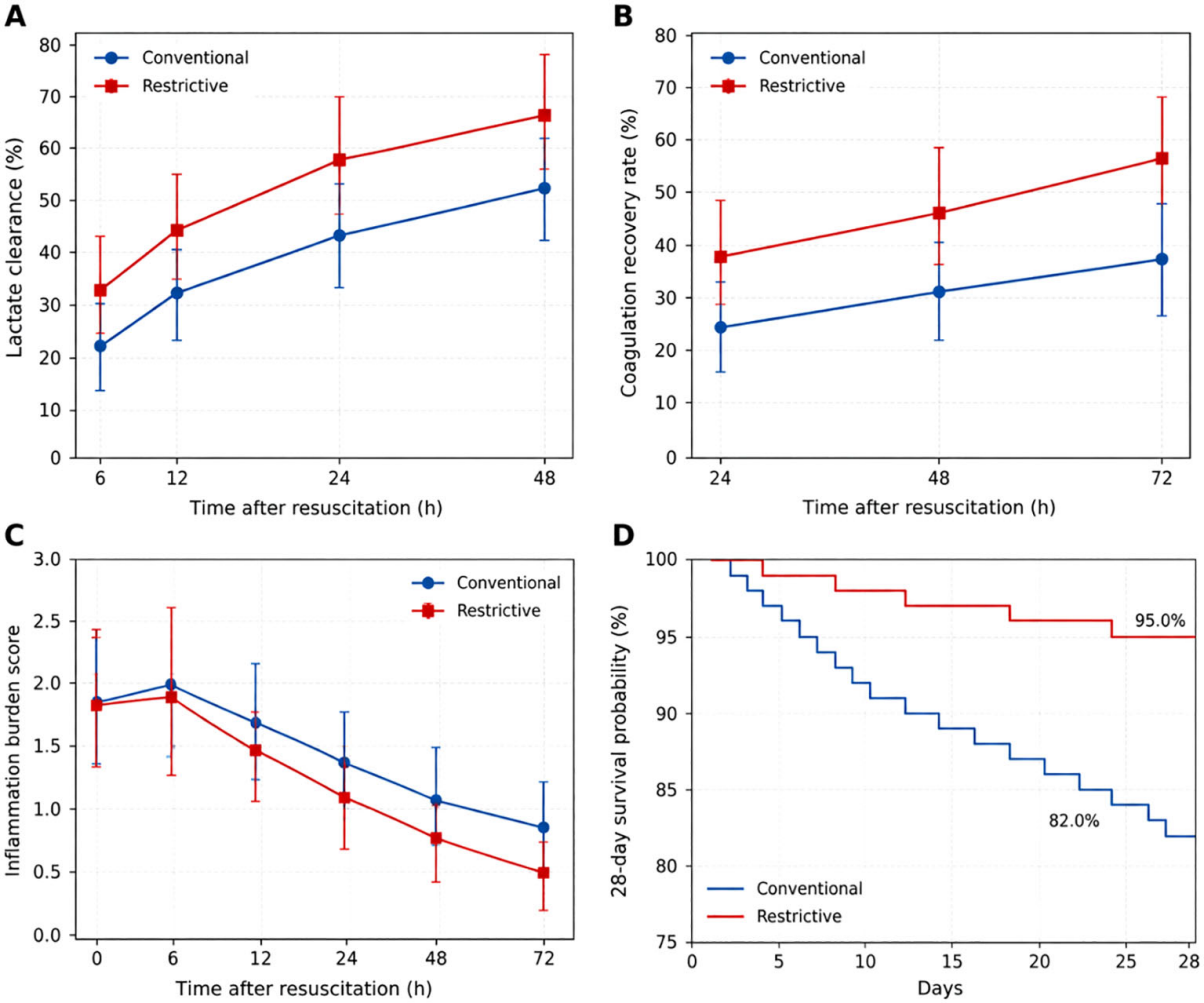
Key recovery and outcome measures. **(A)** Lactate clearance over time. **(B)** Exploratory coagulation recovery rate (CRR) based on serial APTT improvement. **(C)** IBS trajectories. **(D)** Kaplan–Meier curves for 28-day survival.

The restrictive group had a significantly higher 28-day survival rate than the conventional group (95.0% vs. 82.0%, P = 0.003), with early and sustained separation of Kaplan–Meier curves (**Figure 4**). Major complications—including ARDS (16% vs. 34%), DIC (7% vs. 20%), and MODS (15% vs. 36%)—were all significantly less frequent in the restrictive group (all P < 0.01). In this thoracic-dominant trauma population, the reduction in pulmonary and multiorgan complications is clinically notable, as these complications plausibly relate to both hemorrhagic shock severity and resuscitation burden.

SOFA scores decreased more rapidly in the restrictive group over the first 7 days, and the area under the SOFA curve (AUC-SOFA) was significantly lower (28.4 ± 6.7 vs. 36.2 ± 8.3, P < 0.001), indicating a smaller cumulative organ-dysfunction burden (**Table 6**). This difference was directionally consistent with the observed improvements in oxygen metabolism, coagulation, and inflammatory trajectories.

**Table 6:** Clinical outcomes and organ function.

Outcome	Conventional (n = 100)	Restrictive (n = 100)	Effect estimate (95% CI)	P-value
28-day survival, n (%)	82 (82.0)	95 (95.0)	OR = 4.12 (1.48–11.47)	0.003
Complications, n (%)				
- ARDS	34 (34.0)	16 (16.0)	OR = 0.37 (0.19–0.72)	0.003
- DIC	20 (20.0)	7 (7.0)	OR = 0.30 (0.12–0.75)	0.007
- MODS	36 (36.0)	15 (15.0)	OR = 0.31 (0.16–0.62)	<0.001
- AKI	18 (18.0)	11 (11.0)	OR = 0.56 (0.25–1.26)	0.183
Organ function outcomes				
Baseline SOFA	8.4 ± 2.3	8.5 ± 2.4	MD = 0.1 (−0.6 to 0.8)	0.762
Day 3 SOFA	7.2 ± 2.1	6.1 ± 1.9	MD = −1.1 (−1.7 to −0.5)	<0.001
Day 7 SOFA	5.6 ± 1.8	4.3 ± 1.5	MD = −1.3 (−1.8 to −0.8)	<0.001
AUC-SOFA (days 0–7)	36.2 ± 8.3	28.4 ± 6.7	MD = −7.8 (−9.9 to −5.7)	<0.001
Ventilator-free days (28 d)	14.7 ± 5.1	18.4 ± 4.2	MD = 3.7 (2.4–5.0)	<0.001
ICU-free days (28 d)	12.5 ± 5.6	16.2 ± 4.8	MD = 3.7 (2.2–5.2)	<0.001
Hospital-free days (28 d)	8.3 ± 4.9	11.7 ± 5.2	MD = 3.4 (2.0–4.8)	<0.001

Values are mean ± SD or n (%). OR, odds ratio; MD, mean difference; AUC-SOFA, area under the curve of SOFA score (score × days). P-values from t-test, Mann-Whitney U test, χ² test, or Fisher’s exact test as appropriate.

At 28 days, both ventilator-free days (18.4 ± 4.2 vs. 14.7 ± 5.1, P < 0.001) and ICU-free days (16.2 ± 4.8 vs. 12.5 ± 5.6, P < 0.001) were significantly higher in the restrictive group, suggesting more rapid overall recovery and lower short-term critical care utilization.

### 3.6 Adjusted analyses and subgroup analysis

**Figure 5:**
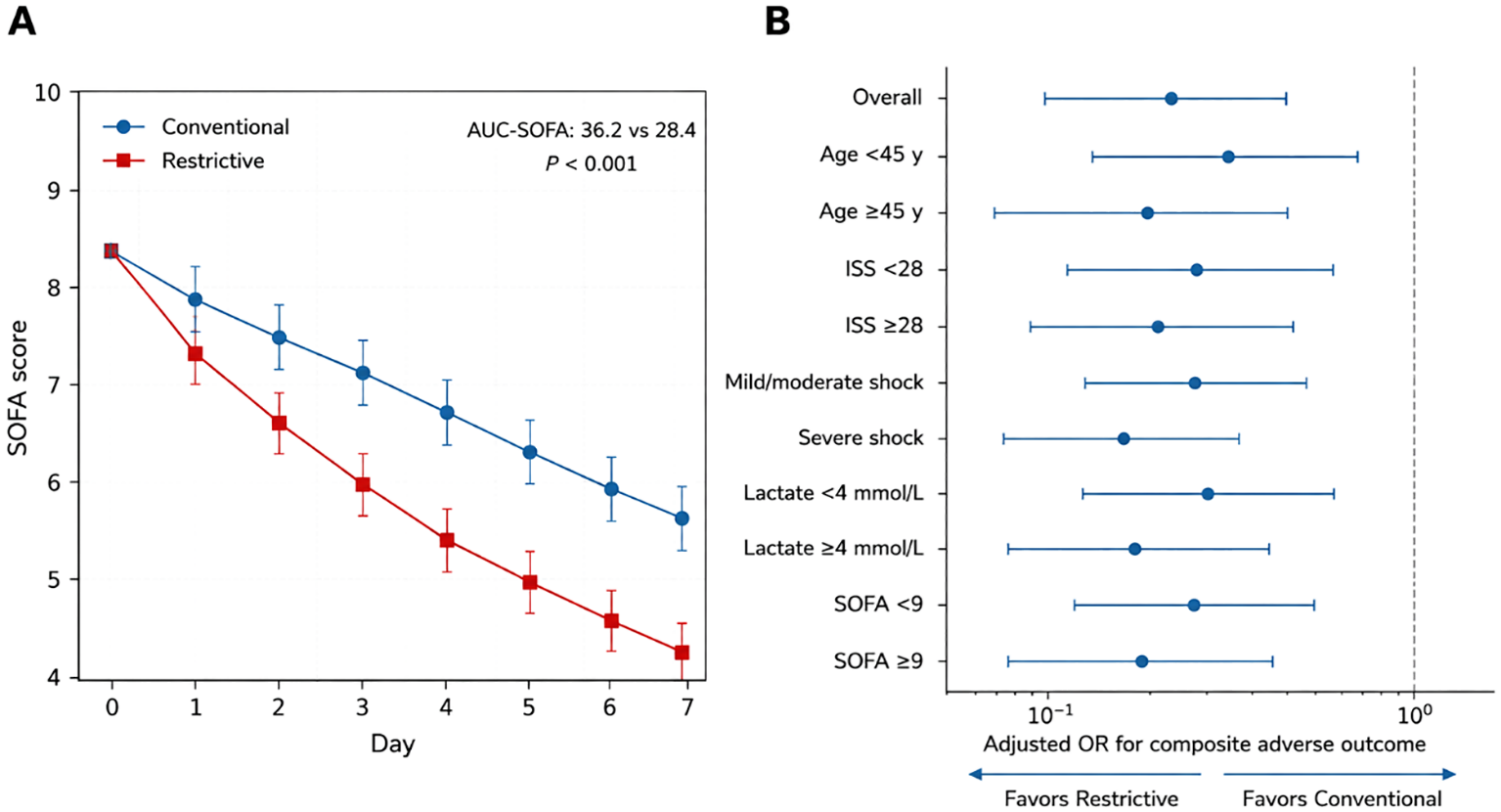
Organ dysfunction and subgroup consistency. **(A)** Dynamic SOFA scores over 7 days. **(B)** Forest plot of subgroup analyses for the composite adverse outcome.

In multivariable Cox regression adjusted for age, ISS, baseline lactate, admission SOFA, and time to admission, restrictive resuscitation remained independently associated with lower 28-day mortality (HR = 0.23, 95% CI 0.09–0.58, P = 0.002). Parallel multivariable logistic regression showed that restrictive resuscitation was independently associated with a lower risk of the composite adverse outcome (OR = 0.24, 95% CI 0.11–0.52, P < 0.001) (**Table 7**). Baseline lactate and ISS were also identified as independent prognostic factors (**Figure 5**). These adjusted analyses should be interpreted as consistency checks rather than proof of causality, but they support the robustness of the primary matched comparisons.

**Table 7:** Multivariable analysis for 28-day mortality and composite adverse outcomes.

Variable	28-day mortality		Composite adverse outcomes	
	HR (95% CI)	P-value	OR (95% CI)	P-value
Restrictive resuscitation (vs. Conventional)	0.23 (0.09–0.58)	0.002	0.24 (0.11–0.52)	<0.001
Age (per 10 years)	1.28 (0.96–1.71)	0.092	1.18 (0.94–1.48)	0.156
ISS (per 5 points)	1.42 (1.08–1.87)	0.012	1.34 (1.07–1.68)	0.011
Baseline lactate (per mmol/L)	1.38 (1.12–1.70)	0.003	1.32 (1.08–1.61)	0.008
Admission SOFA (per point)	1.21 (1.03–1.42)	0.021	1.18 (1.02–1.36)	0.024
Time to admission (per hour)	1.15 (0.92–1.44)	0.218	1.09 (0.89–1.33)	0.404

HR, hazard ratio (Cox regression); OR, odds ratio (logistic regression). Composite adverse outcome, ARDS, DIC, MODS, or death within 28 days. Models adjusted for age, ISS, baseline lactate, admission SOFA, and time to admission. Proportional hazards assumption verified (global P = 0.18).

Forest-plot subgroup analyses showed that the direction of association generally favored restrictive resuscitation across predefined strata. However, in patients with isolated pulmonary contusion without hemothorax (n=28), the association between restrictive resuscitation and the composite adverse outcome was substantially attenuated and not statistically significant (OR = 0.68, 95% CI 0.21-2.15, P = 0.51). Although the confidence interval is wide and compatible with either benefit or harm, this null finding suggests that the apparent benefit of the restrictive strategy may be less pronounced or absent in specific injury phenotypes where respiratory compromise, rather than hemorrhagic shock, is the dominant pathophysiological driver.

### 3.7 Sensitivity analyses

Complete-case analysis (n = 156) yielded effect estimates similar to the primary mixed-model analysis (DO_2_ at 72 h: mean difference 72.3 vs. 68.5 mL/min, P < 0.001; APTT at 72 h: −3.2 vs. −3.0 s, P = 0.002). Worst-case imputation attenuated but did not eliminate between-group differences (DO_2_: mean difference 38.6 mL/min, P = 0.042; APTT: −2.1 s, P = 0.038). Multiple imputation using chained equations yielded results nearly identical to the primary analysis. These findings suggest that the observed physiological differences are unlikely to be fully explained by missing data or survivor bias, although some attenuation in the worst-case scenario indicates that differential early mortality may contribute to the magnitude of the observed differences. In the survivors-only analysis (n=177, comprising 95 restrictive and 82 conventional patients), the restrictive group continued to demonstrate significantly better physiological profiles at 72 hours, including higher DO_2_ (596.1 ± 60.8 vs. 515.8 ± 58.7 mL/min, P < 0.001), shorter APTT (32.1 ± 4.2 vs. 36.8 ± 4.7 s, P < 0.001), and lower IL-6 (27.2 ± 10.8 vs. 40.5 ± 14.1 pg/mL, P < 0.001) compared with the conventional group. This survivors-only analysis suggests that the observed physiological advantages are not solely explained by differential early mortality but represent a true association with the resuscitation strategy, although residual bias cannot be entirely excluded.

Three alternative approaches—1:2 matching, stricter caliper (0.1), and matching with replacement—all yielded hazard ratios for 28-day mortality ranging from 0.21 to 0.26 (all P < 0.01), supporting the robustness of the primary matching results.

To assess the robustness of the primary findings against potential confounding, we performed several sensitivity analyses. First, multivariable models adjusting for trauma severity indices (RTS, TRISS, Chest AIS, PaO_2_/FiO_2_ ratio) and co-interventions (transfusion variables, TXA use) yielded results consistent with the primary analysis (for 28-day mortality: adjusted HR = 0.24, 95% CI 0.10–0.61, P = 0.002; for composite adverse outcome: adjusted OR = 0.25, 95% CI 0.12–0.54, P < 0.001). Additionally, separate models adjusting for the excluded baseline severity indices—including fibrinogen, base deficit trajectory, RTS, TRISS, Chest AIS, and PaO_2_/FiO_2_ ratio—also showed similar results (adjusted HR = 0.24, 95% CI 0.09–0.63, P = 0.003). Inverse probability weighting (HR = 0.25, 95% CI 0.10–0.62, P = 0.003), multiple imputation for missing baseline variables (OR = 0.26, 95% CI 0.12–0.55), and exclusion of patients with extreme fluid volumes (>4000 mL, n = 14) all yielded consistent estimates, suggesting that the findings are not driven by outliers or the specific adjustment method. Second, to quantify the potential impact of unmeasured confounding, we calculated the E-value for the primary Cox regression result (HR = 0.23). The E-value was 4.5 for the point estimate and 2.9 for the lower confidence limit of 0.09. This implies that an unmeasured confounder would need to be associated with both the resuscitation strategy and 28-day mortality by a risk ratio of at least 4.5 to completely negate the observed survival benefit—a magnitude of association that is substantial and unlikely to be present in this clinical context. However, while these sensitivity analyses strengthen the credibility of our findings, they cannot completely exclude residual or unmeasured confounding.

### 3.8 Correlation analysis and exploratory mechanistic signals

**Figure 6:**
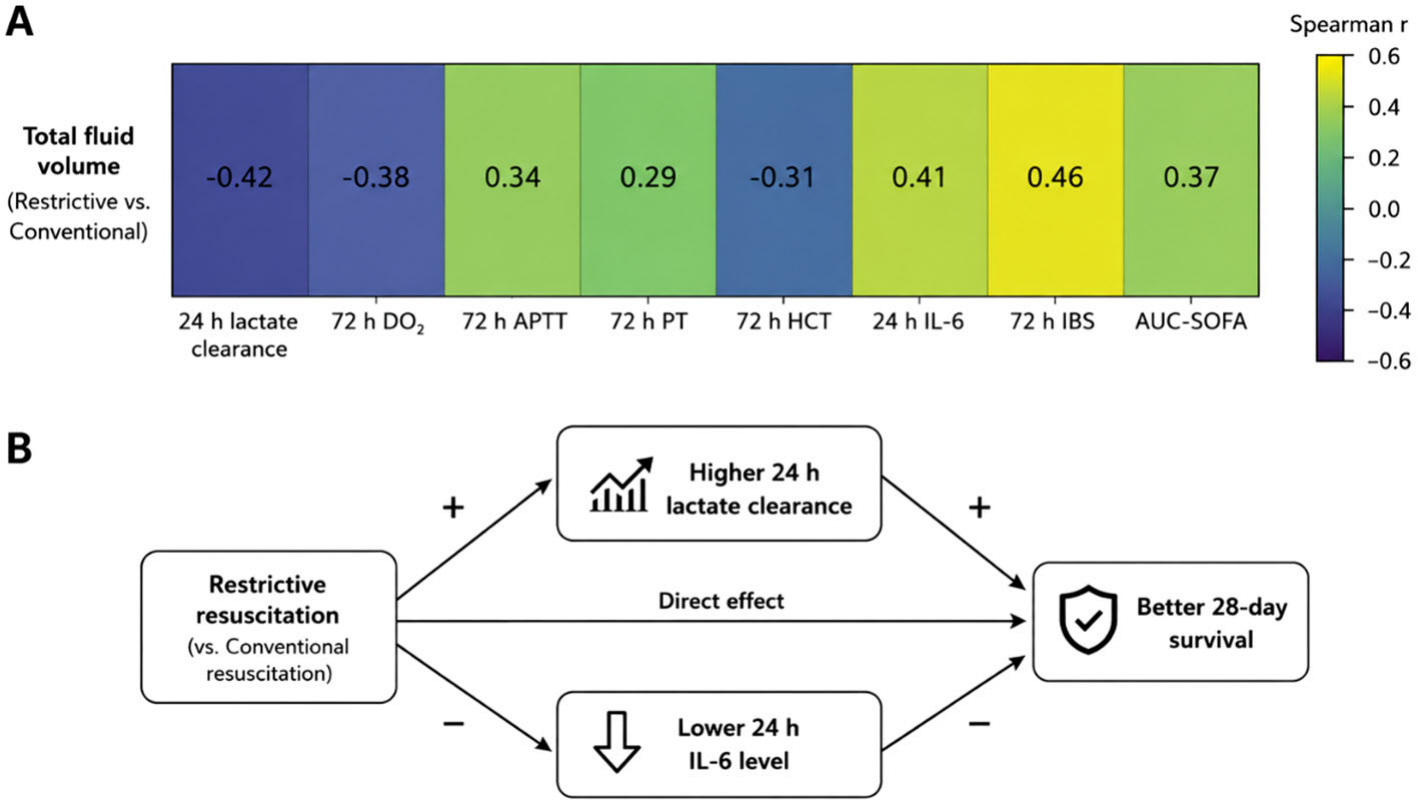
Exploratory correlation and mediation analyses. **(A)** Heatmap of Spearman correlations between total fluid volume and key post-resuscitation parameters. **(B)** Exploratory mediation diagram showing the potential intermediary roles of 24 h lactate clearance and 24 h IL-6 in the association between restrictive resuscitation and 28-day survival.

Spearman correlation analysis revealed that higher total fluid volume was positively correlated with inflammatory burden (IL-6: r = 0.42, P < 0.001; TNF-α: r = 0.38, P < 0.001) and prolonged coagulation parameters (APTT: r = 0.45, P < 0.001; PT: r = 0.39, P < 0.001), and negatively correlated with HCT (r = −0.48, P < 0.001) and DO_2_ (r = −0.41, P < 0.001). No significant correlation was found between total fluid volume and IL-10 levels (r = 0.08, P = 0.31). These associations remained directionally similar after adjustment for baseline severity (**Figure 6A**).

Exploratory mediation analysis suggested that 24-h lactate clearance and 24-h IL-6 levels may partially mediate the association between restrictive resuscitation and 28-day survival. However, because treatment allocation was not randomized and mediators were measured within an observational framework, these findings should be interpreted as hypothesis-generating rather than causal proof (**Figure 6B**).

## 4 Discussion

This propensity score−matched cohort study of 200 patients with severe chest trauma and hemorrhagic shock found that a DCR−like restrictive strategy—characterized by limited crystalloid, permissive hypotension, and early blood product use—was associated with better outcomes and more favorable physiological trajectories than conventional resuscitation. The restrictive group received 30.3% less fluid with similar resuscitation times and higher fluid efficiency (FER: 13.2 vs. 8.5 mmHg/L, P < 0.001). Groups were balanced for RTS, TRISS, Chest AIS, and key physiologic parameters, with no differences in transfusion volumes or ratios. Nevertheless, because the restrictive strategy bundles multiple DCR components, its independent contribution cannot be isolated.

The restrictive group showed faster improvements in SvO_2_, DO_2_, VO_2_, and lactate clearance, with a 33.4% lower ODI. These associations are biologically plausible: excessive crystalloid dilutes hemoglobin, reduces oxygen−carrying capacity, and may exacerbate interstitial edema that impairs tissue oxygen diffusion (Weng et al., 2019; Zhu et al., 2022; Yao et al., 2024). However, improved hematocrit and DO_2_ could reflect RBC transfusion rather than fluid restriction. Although total RBC volumes did not differ between groups (median 2.0 units in both), we cannot exclude that transfusion timing, hemoglobin thresholds, or component sequencing contributed to the observed trajectories. Sensitivity analyses adjusting for transfusion and MTP activation yielded consistent results.

The restrictive group exhibited more rapid APTT, PT, and HCT recovery, with higher CRR at 72 h. These findings align with trauma−induced coagulopathy pathophysiology, where dilution of coagulation factors plays a central role (Holcomb et al., 2015; Jang et al., 2022). Large−volume crystalloid may exacerbate TIC through direct dilution, reduced fibrinogen, platelet dysfunction, and hypothermia (Wang et al., 2020; Zhang et al., 2023). However, improvements in PT, APTT, and CRR, and the lower DIC rate, could reflect plasma, platelet, fibrinogen, or cryoprecipitate administration rather than reduced crystalloid alone. We lacked data on transfusion timing and viscoelastic−guided therapy; thus, coagulation findings cannot be mechanistically attributed to this strategy.

IL−6, IL−10, and TNF−α declined more steeply in the restrictive group, consistent with evidence that excessive crystalloid damages the endothelial glycocalyx and triggers systemic inflammation (Shao et al., 2019b; Chi et al., 2022). By reducing fluid load, restrictive resuscitation may attenuate pro−inflammatory cytokine release (Yang et al., 2021; Jiang et al., 2023). Given the observational design and post−hoc IBS derivation, these findings are hypothesis−generating.

The restrictive group had significantly higher 28-day survival (95.0% vs. 82.0%) and lower ARDS, DIC, and MODS, with more ventilator-free and ICU-free days. These findings align with previous studies linking restrictive fluid strategies to improved outcomes in various shock states, including septic shock and ARDS (Hjortrup et al., 2017; Esper Treml et al., 2023; Hu et al., 2023; Jiang et al., 2023). The observed 13% absolute survival difference favoring the restrictive strategy is substantial and should be interpreted with extreme caution. While our E-value analysis suggested robustness against moderate unmeasured confounding, the possibility of residual selection bias cannot be dismissed. Despite rigorous PSM, the decision to allocate a patient to a restrictive strategy was not random and may have been influenced by unrecorded clinician gestalt, such as perceived physiological reserve or anticipated responsiveness to hemostatic intervention. Such unmeasured factors could have systematically favored the restrictive group, potentially inflating the observed effect size. Furthermore, the survival benefit was derived from a single-center cohort, and its magnitude is larger than what is typically reported in similar trauma populations, suggesting that our findings may represent an overestimate of the true effect. Therefore, this survival signal should be viewed as hypothesis-generating and warrants confirmation in a prospective, multicenter randomized trial.

Our findings are consistent with recent reports by Li et al. (2026) and Zheng et al. (2023), who observed improved coagulation and inflammatory profiles with restrictive resuscitation in traumatic shock. However, our study extends these observations by providing serial multi-time-point physiological measurements specifically in a chest-dominant trauma cohort. Bickell et al. (1994) showed that delayed restricted fluid improved survival in penetrating torso injury. Shao et al. (2019b) found that lower MAP (65–70 mmHg) during resuscitation suppressed pro-inflammatory cytokines. In septic shock, Jiang et al. (2023) reported lower mortality and organ dysfunction with restrictive resuscitation, while Hjortrup et al. (2017) showed improved circulatory efficacy. Esper Treml et al. (2023) observed that restrictive fluid balance improved 28-day survival in ventilated ARDS patients. Our study extends this literature by providing multi-time-point dynamic monitoring of oxygen metabolism, coagulation, and inflammation specifically in chest-dominant trauma. To our knowledge, this is one of the few studies with such comprehensive serial physiological data in severe chest trauma with hemorrhagic shock. We also introduced three exploratory composite metrics—ODI, CRR, and IBS—that summarize complex physiological trajectories. Our pre-specified exploratory mediation analysis suggested that 24-h lactate clearance and 24-h IL-6 may partially mediate the association between restrictive resuscitation and 28-day survival.

A critical issue is whether observed benefits reflect DCR-like restrictive resuscitation strategy per se or the broader DCR bundle. The restrictive group received not only less crystalloid but also earlier blood products and permissive hypotension—core DCR elements (Bickell et al., 1994; Holcomb et al., 2015). The conventional group received crystalloid-predominant resuscitation with blood products triggered by hemoglobin thresholds. Thus, observed differences likely represent the combined effect of multiple DCR components rather than isolated fluid reduction. If benefit derives from the entire bundle, implementing DCR-like restrictive resuscitation strategy alone—without permissive hypotension and early blood products—may not reproduce the favorable outcomes. Factorial or sequential trials are needed to disentangle independent contributions. We therefore frame conclusions as strategy-level associations, not effects of DCR-like restrictive resuscitation strategy alone.

A related critical issue is potential confounding of physiological endpoints by blood product administration. Hematocrit, DO_2_, VO_2_, PT, APTT, DIC, and coagulation recovery are all strongly influenced by transfusion practice. Higher hematocrit and DO_2_ may reflect RBC transfusion; improved PT/APTT and lower DIC may reflect plasma, platelet, fibrinogen, or cryoprecipitate. Although total transfusion volumes and ratios were balanced, we cannot determine whether transfusion timing or component sequencing differed between groups—both could substantially influence physiological trajectories. Viscoelastic assays were not standard, and we lack fibrinogen/cryoprecipitate dosing and timing data. Consequently, physiological results cannot be mechanistically interpreted as evidence that DCR-like restrictive resuscitation improved oxygen metabolism, coagulation, or inflammation. Observed associations may reflect combined effects of DCR-like restrictive resuscitation strategy, transfusion practices, and other unmeasured DCR components.

The three composite metrics—ODI, CRR, and IBS—are novel, post-hoc, and internally derived. Several caveats must be emphasized. First, ODI assumes any DO_2_ below age-predicted normal represents a deficit and that deficits at different time points contribute equally—assumptions not validated in trauma. Second, CRR relies exclusively on APTT and does not account for platelet function, fibrinogen, or other factors; it has not been validated against viscoelastic assays. Third, IBS assumes three inflammatory markers are adequately represented by a single principal component—a simplification that may not capture the full inflammatory complexity. Fourth, all three metrics were developed and tested in the same cohort, raising overfitting risk. We caution against clinical application of these indices until independently validated in prospective, multicenter datasets with predefined analytical plans.

Key strengths include propensity score matching, detailed fluid protocols with explicit hemodynamic targets, multi-time-point serial measurements, transparent missing data handling (overall 7.4%), and comprehensive matching diagnostics (C-statistic = 0.71, post-matching SMDs ≤0.09). Consistency across multiple physiological domains and sensitivity analyses strengthens credibility.

Several limitations should be acknowledged. First, despite PSM and sensitivity analyses, residual confounding from unmeasured variables cannot be excluded. Most importantly, although we collected total transfusion volumes, MTP activation, TXA use, and blood product ratios—with no significant between-group differences—we lacked detailed data on transfusion timing, component sequencing, and viscoelastic-guided therapy. These missing data are critical because timing and sequence can profoundly influence coagulation, oxygen delivery, and inflammation independently of fluid volume. Fibrinogen and cryoprecipitate use were captured only as binary variables without dosing/timing details. This granularity gap substantially limits attribution of physiological improvements to DCR-like restrictive resuscitation strategy versus transfusion management. Second, this was a single-center study, limiting generalizability. Third, sample size (200 matched patients) may be insufficient for small effect sizes or extensive subgroup analyses. Fourth, follow-up was limited to 28 days; longer-term outcomes were not assessed. Fifth, SvO_2_ was substituted with ScvO_2_ in some patients, introducing possible measurement discrepancies. Sixth, lack of viscoelastic assays limited comprehensive coagulation assessment. Seventh, the three composite metrics—ODI, CRR, and IBS—were developed post-hoc and not externally validated. Eighth, the observed 13% absolute survival difference is large for a retrospective study; residual confounding or selection bias cannot be ruled out. Ninth, serial physiological analyses are susceptible to survivor bias. Patients who died before 72 h contributed data only up to their last time point, and higher mortality in the conventional group (18% vs. 5%) caused greater attrition of sicker patients at later time points. Although we used linear mixed-effects models and sensitivity analyses including worst-case imputation, we cannot fully exclude survivor bias. Worst-case imputation showed attenuation of between-group differences, suggesting some observed physiological advantage in the restrictive group may reflect differential survival rather than a true strategy effect.

Consistent associations across multiple physiological domains support the hypothesis that a DCR-like restrictive resuscitation strategy may be associated with clinical benefits in severe chest trauma with hemorrhagic shock. The enhanced association in sicker patients (higher ISS, higher lactate, or higher SOFA) suggests this subgroup warrants particular attention. However, given the observational design and potential for residual confounding, these findings should not be interpreted as sufficient evidence for immediate protocol change. Instead, they highlight the critical need for multicenter prospective randomized controlled trials with predefined restrictive versus liberal protocols, standardized co-intervention reporting (transfusion ratios, TXA, hemostatic procedures), and long-term outcome assessment. The REFILL study in obstetric hemorrhage (de Lange et al., 2018) and Boulet et al.’s pilot trial in septic shock (Boulet et al., 2024) demonstrate feasibility of prospective randomized designs in critically ill populations. An important exploratory finding is the null result in the isolated pulmonary contusion subgroup. This may have several explanations. First, this subgroup had limited statistical power (n=28), and the wide confidence interval precludes definitive conclusions. Second, patients with isolated contusions might have less profound hemorrhagic shock, and thus the benefits of reducing crystalloid volume to improve oxygenation might be offset by the need for adequate perfusion to the contused lung. Third, excessive fluid restriction in this specific population could theoretically promote atelectasis or impair clearance of inflammatory exudates. This hypothesis-generating finding underscores that a one-size-fits-all restrictive strategy may not be appropriate for all chest trauma patients and highlights the need for personalized resuscitation approaches based on injury pattern. Future trials should stratify by injury phenotype to explore this differential response. Future studies should incorporate viscoelastic assays, continuous tissue oxygenation monitoring, and real-time lactate clearance tracking to refine resuscitation endpoints.

## 5 Conclusions

In this propensity score-matched retrospective cohort study, a DCR-like restrictive resuscitation strategy—incorporating limited crystalloid, permissive hypotension, and early blood product use—was associated with improved oxygen metabolism, accelerated coagulation recovery, attenuated inflammation, higher 28-day survival, and fewer complications than conventional crystalloid-predominant resuscitation.

Several caveats warrant emphasis. The observed physiological improvements may reflect blood component therapy rather than fluid restriction per se, as we lacked granular data on transfusion timing and component sequencing. Additionally, because the restrictive strategy bundles multiple DCR components, its benefits cannot be ascribed solely to fluid limitation. Finally, the observational, single-center design precludes causal inference, and the survival difference may be confounded by unmeasured factors.

These findings support prospective, multicenter randomized trials with standardized documentation of transfusion practices to establish whether restrictive fluid therapy confers true causal benefit. For now, clinical decisions should be guided by individual patient assessment and existing DCR principles. This study provides a foundation for hypothesis generation rather than a rationale for practice change.

## Data Availability

The raw data supporting the conclusions of this article will be made available by the authors, without undue reservation.

## Supplementary material

Supplementary Table 1 Comparison of conventional and restrictive resuscitation protocols. TBI, traumatic brain injury; MAP, mean arterial pressure; SBP, systolic blood pressure; Hb, hemoglobin.

## References

[B1] Anker A. M. Ruewe M. Prantl L. Baringer M. Pawlik M. T. Zeman F. . (2024). Biomarker-guided acute kidney injury risk assessment under liberal versus restrictive fluid therapy - the prospective-randomized MAYDAY-trial. Sci. Rep. 14, 17094. doi: 10.1038/s41598-024-68079-2 39048691 PMC11269689

[B2] (2008). Guidelines for resuscitation of hypovolemic shock (2007). Zhongguo Wei Zhong Bing Ji Jiu Yi Xue 20, 129–134. doi: 10.1016/b978-1-4377-1367-1.00191-9 18328122

[B3] Bickell W. H. Wall M. J. Jr. Pepe P. E. Martin R. R. Ginger V. F. Allen M. K. . (1994). Immediate versus delayed fluid resuscitation for hypotensive patients with penetrating torso injuries. N. Engl. J. Med. 331, 1105–1109. doi: 10.1016/0300-9572(95)90705-m 7935634

[B4] Boulet N. Quenot J. P. Serrand C. Antier N. Garnier S. Buzancais A. . (2024). Impact on fluid balance of an optimized restrictive strategy targeting non-resuscitative fluids in intensive care patients with septic shock: a single-blind, multicenter, randomized, controlled, pilot study. Crit. Care 28, 429. doi: 10.1186/s13054-024-05155-z 39709493 PMC11663312

[B5] Boyd C. R. Tolson M. A. Copes W. S. (1987). Evaluating trauma care: the TRISS method. Trauma Score and the Injury Severity Score. J. Trauma 27, 370–378. 3106646

[B6] Bu Y. Z. Liu X. Z. Zhou T. N. Liu X. D. Jin H. X. Liu X. J. . (2022). Clinical characteristics and diagnosis and treatment strategies of patients with severe traumatic aortic injury. Zhonghua Xin Xue Guan Bing Za Zhi 50, 767–773. doi: 10.3760/cma.j.cn112148-20220430-00333 35982008

[B7] Champion H. R. Sacco W. J. Copes W. S. Gann D. S. Gennarelli T. A. Flanagan M. E. (1989). A revision of the trauma score. J. Trauma 29, 623–629. doi: 10.1097/00005373-198905000-00017 2657085

[B8] Chi Y. Jiang X. Chai J. Chang Y. Liu T. Liu X. . (2022). Protective effect of restrictive resuscitation on vascular endothelial glycocalyx in pigs with traumatic hemorrhagic shock. Ann. Transl. Med. 10, 177. doi: 10.21037/atm-21-7004 35280352 PMC8908128

[B9] de Lange N. Schol P. Lancé M. Woiski M. Langenveld J. Rijnders R. . (2018). Restrictive Versus Massive Fluid Resuscitation Strategy (REFILL study), influence on blood loss and hemostatic parameters in obstetric hemorrhage: study protocol for a randomized controlled trial. Trials 19, 166. doi: 10.1186/s13063-018-2512-z 29510717 PMC5838856

[B10] Ebihara T. Shimizu K. Ojima M. Nakamura Y. Mitsuyama Y. Ohnishi M. . (2022). Energy expenditure and oxygen uptake kinetics in critically ill elderly patients. JPEN J. Parenter Enteral Nutr. 46, 75–82. doi: 10.1002/jpen.2098 33704803

[B11] Elkbuli A. Kinslow K. Sen-Crowe B. Liu H. McKenney M. Ang D. (2021). Outcomes of resuscitative endovascular balloon occlusion of the aorta (REBOA) utilization in trauma patients with and without traumatic brain injuries: A national analysis of the American College of Surgeons Trauma Quality Improvement Program data set. Surgery 170, 284–290. doi: 10.1016/j.surg.2021.01.043 33676729

[B12] Esper Treml R. Caldonazo T. Filho P. H.A. Mori A. L. Carvalho A. S. Serrano J. S.F. . (2023). Effect of restrictive cumulative fluid balance on 28-day survival in invasively ventilated patients with moderate to severe ARDS due to COVID-19. Sci. Rep. 13, 18504. doi: 10.1038/s41598-023-45483-8 37898681 PMC10613222

[B13] Hjortrup P. B. Haase N. Wetterslev J. Lange T. Bundgaard H. Rasmussen B. S. . (2017). Effects of fluid restriction on measures of circulatory efficacy in adults with septic shock. Acta Anaesthesiol Scand. 61, 390–398. doi: 10.1111/aas.12862 28150304

[B14] Holcomb J. B. Tilley B. C. Baraniuk S. Fox E. E. Wade C. E. Podbielski J. M. . (2015). Transfusion of plasma, platelets, and red blood cells in a 1:1:1 vs a 1:1:2 ratio and mortality in patients with severe trauma: the PROPPR randomized clinical trial. Jama 313, 471–482. doi: 10.1001/jama.2015.12 25647203 PMC4374744

[B15] Hu X. Zhang J. Wang P. Dai X. (2023). Practice and effect evaluation of early restrictive fluid resuscitation strategy in the nursing care of patients with sepsis in the emergency department: a retrospective cohort study. JBI Evid Implement 21, 269–276. doi: 10.1097/xeb.0000000000000365 36917161

[B16] Jang J. Y. Bae K. S. Chang S. W. Jung K. Kim D. H. Kang B. H. (2022). Current management and clinical outcomes for patients with haemorrhagic shock due to pelvic fracture in Korean regional trauma centres: A multi-institutional trial. Injury 53, 488–495. doi: 10.1016/j.injury.2021.12.015 34916034

[B17] Jiang Z. Luo F. Liu Y. Sun X. Tan G. Chen Z. . (2023). Restrictive fluid resuscitation in septic shock patients has lower mortality and organ dysfunction rates than standard therapy. Shock 60, 739–745. doi: 10.1097/shk.0000000000002235 37962948

[B18] Jiang Z. Liu Y. Ren J. (2020). The application progress of fluid de-escalation therapy in abdominal infection-induced septic shock. Zhonghua Wei Zhong Bing Ji Jiu Yi Xue 32, 1403–1408. doi: 10.3760/cma.j.cn121430-20200714-00519 33463507

[B19] Li L. Zhou J. Song K. Liu H. Wang X. Zhu Y. . (2026). Application of restrictive fluid resuscitation in emergency traumatic hemorrhagic shock and impact on blood gas indicators. Ann. Vasc. Surg. 122, 352–357. doi: 10.1016/j.avsg.2025.06.009 40541774

[B20] Mekontso Dessap A. AlShamsi F. Belletti A. De Backer D. Delaney A. Møller M. H. . (2025). European Society of Intensive Care Medicine (ESICM) 2025 clinical practice guideline on fluid therapy in adult critically ill patients: part 2-the volume of resuscitation fluids. Intensive Care Med. 51, 461–477. doi: 10.1007/s00134-025-07840-1 40163133

[B21] Mitchnik I. Y. Talmy T. Radomislensky I. Chechik Y. Shlaifer A. Almog O. . (2022). Femur fractures and hemorrhagic shock: Implications for point of injury treatment. Injury 53, 3416–3422. doi: 10.1016/j.injury.2022.08.053 36041921

[B22] Qian J. Zhang J. (2024). Establishing a prognostic prediction model for patients with septic shock based on the completion time of fluid resuscitation and the negative fluid balance volumes. Zhonghua Wei Zhong Bing Ji Jiu Yi Xue 36, 244–248. doi: 10.3760/cma.j.cn121430-20240102-00001 38538351

[B23] Shao Z. Du Z. Wang R. . (2019a). Zhonghua Wei Zhong Bing Ji Jiu Yi Xue 31, 428–433. doi: 10.3760/cma.j.issn.2095-4352.2019.04.011 31109415

[B24] Shao Z. Du Z. Wang R. Wang Z. He X. Wang H. . (2019b). Effects of different target blood pressure resuscitation on peripheral blood inflammatory factors and hemodynamics in patients with traumatic hemorrhagic shock. Zhonghua Wei Zhong Bing Ji Jiu Yi Xue 31, 428–433. doi: 10.3760/cma.j.issn.2095-4352.2019.04.011 31109415

[B25] Taniguchi K. Kaneko T. Yamaguchi T. (2025). Delayed hemorrhagic shock due to reverse chance thoracic vertebrae fracture complicated with hypoxemia caused by diaphragmatic eventration. Trauma Case Rep. 57, 101177. doi: 10.1016/j.tcr.2025.101177 40276082 PMC12019419

[B26] Wagner R. B. Crawford W. J. Schimpf P. P. (1988). Classification of parenchymal injuries of the lung. Radiology 167, 77–82. doi: 10.1148/radiology.167.1.3347751 3347751

[B27] Wang W. Feng Q. Yang W. Liang Y. Li Z. Wang H. (2020). Effect of different fluid resuscitation strategies on renal function in patients with septic shock induced acute kidney injury. Zhonghua Wei Zhong Bing Ji Jiu Yi Xue 32, 1080–1084. doi: 10.3760/cma.j.cn121430-20200717-00529 33081894

[B28] Weng C. Lan K. Li T. Zhang L. Wang J. Lai X. (2019). Regional hypothermia attenuates secondary-injury caused by time-out application of tourniquets following limb fragments injury combined with hemorrhagic shock. Scand. J. Trauma Resusc Emerg. Med. 27, 104. doi: 10.1186/s13049-019-0678-3 31752982 PMC6873525

[B29] Yang M. Dai X. H. Guo G. H. Min D. H. Liao X. C. Zhang H. Y. . (2021). Fluid resuscitation strategy and efficacy evaluation in shock stage in severely burned children with different burn areas in different age groups. Zhonghua Shao Shang Za Zhi 37, 929–936. doi: 10.3760/cma.j.cn501120-20210408-00119 34689462 PMC11917242

[B30] Yao Z. Chen Y. Li D. Li Y. Liu Y. Fan H. (2024). Hemorrhagic shock assessed by tissue microcirculatory monitoring: A narrative review. Shock 61, 509–519. doi: 10.1097/SHK.0000000000002242 37878487

[B31] Zhang H. Zhao J. Zhang Y. Liu D. Hu B. Wang H. . (2022). Clinical effect of fluid resuscitation guided by intra-abdominal pressure and oxygenation index for severe acute pancreatitis patients. Zhonghua Wei Zhong Bing Ji Jiu Yi Xue 34, 525–528. doi: 10.1097/SHK.0000000000002242 35728856

[B32] Zhang C. Ou Y. Qian H. Xu Y. (2023). Effect of early fluid balance on the prognosis in severe acute pancreatitis. Zhonghua Wei Zhong Bing Ji Jiu Yi Xue 35, 524–527. doi: 10.3760/cma.j.cn121430-20221020-00933 37308235

[B33] Zheng J. Zhu J. Cao L. Dong M. Mao Y. Zhao Z. . (2023). Effect of restrictive fluid resuscitation on the coagulation function and hemodynamic parameters in patients with hemorrhagic traumatic shock. Clinics (Sao Paulo) 78, 100300. doi: 10.1016/j.clinsp.2023.100300 37931530 PMC10654136

[B34] Zhu Y. Ma S. Deng H. Y. Wu Y. Zhang J. Xiang X. M. . (2022). The characteristics of organ function damage of hemorrhagic shock in hot environment and the effect of hypothermic fluid resuscitation. Shock 57, 526–535. doi: 10.1097/shk.0000000000001873 34628454

