# Supplementary material for: DCR-like restrictive resuscitation is associated with enhanced oxygen metabolism, coagulation recovery, and reduced inflammation in severe chest trauma and shock
Source: Front Physiol. 2026 Jul 15;17:1827424. doi: 10.3389/fphys.2026.1827424 (PMC13414272; doi:10.3389/fphys.2026.1827424)
Supplement: Supplementary Table 1 — Comparison of conventional and restrictive resuscitation protocols. TBI, traumatic brain injury; MAP, mean arterial pressure; SBP, systolic blood pressure; Hb, hemoglobin. [file Table1.docx]

**Supplementary Table 1. Comparison of conventional and restrictive resuscitation protocols**

| Parameter | Conventional resuscitation | Restrictive resuscitation |
| --- | --- | --- |
| Target MAP (mmHg) | ≥65 | 50–65 (permissive hypotension) |
| Target SBP (mmHg) | ≥90 | 70–90 |
| Initial crystalloid bolus | 500–1000 mL over 15–30 min | 250 mL only if MAP <50 mmHg |
| Max crystalloid before hemostasis | No predefined limit | ≤1000 mL total |
| Blood product priority | After crystalloid, triggered by Hb <7 g/dL | Early, before large crystalloid; trigger Hb <8 g/dL |
| Colloid use | Permitted (clinician discretion) | Discouraged |
| Resuscitation endpoint | MAP ≥65 mmHg sustained | Surgical hemostasis or spontaneous MAP >65 mmHg |
| Exclusion for TBI | Not applicable (TBI not excluded from conventional group) | MAP must be ≥80 mmHg if TBI suspected (then not eligible for restrictive strategy) |

TBI: traumatic brain injury; MAP: mean arterial pressure; SBP: systolic blood pressure; Hb: hemoglobin.
